# Supplementary material for: Health and well-being needs of Indigenous adolescents: a protocol for a scoping review of qualitative studies
Source: BMJ Open. 2024 May 20;14(5):e079942. doi: 10.1136/bmjopen-2023-079942 (PMC11110593; doi:10.1136/bmjopen-2023-079942)
Supplement: Supplementary data [file bmjopen-2023-079942supp001.pdf]

# SUPPLEMENTARY FILE 1: SEARCH TERMS

*Table 1. Search terms for the Pubmed, CINAHL, Embase, Scopus and Informit databases. To use, copy the entire contents of each cell into the search box of the relevant database. After the contents of each box has been searched separately, combine queries using the AND operator.*

| **Search filters (combine with AND)** | **Terms (Pubmed)** | **Terms (CINAHL)** | **Terms (Embase)** | **Terms (Scopus)** | **Terms (Informit Indigenous and Health Collections)** |
|---|---|---|---|---|---|
| **Indigenous peoples of eligible geographical locations:** | (American Indian*[tiab] OR<br>Native American*[tiab] OR<br>First Nation [tiab] OR<br>First Nations [tiab] OR<br>Inuit*[tiab] OR<br>Greenlandic [tiab] OR<br>Metis*[tiab] OR<br>Two spirit* [tiab] OR<br>Two-spirit* [tiab] OR<br>Kanaka Maoli* [tiab] OR<br>Maori*[tiab] OR<br>Maaori*[tiab] OR<br>Aborigin*[tiab] OR<br>Torres Strait Islander*[tiab] OR<br>Sami*[tiab] OR<br>Saami* [tiab] OR<br>American Native Continental Ancestry Group [MH:noexp] OR<br>Indians, North American [MH] OR<br>Indigenous Canadians [MH] OR<br>Inuits [MH] OR<br>"Native Hawaiian or other Pacific Islander" [MH])<br><br>OR<br><br>(<br>(Native*[tiab] OR<br>Indigenous*[tiab] OR | (“American Indian*” OR<br>“Native American*” OR<br>“First Nation” OR<br>“First Nations” OR<br>Inuit* OR<br>Greenlandic OR<br>Metis* OR<br>“Two spirit*” OR<br>Two-spirit* OR<br>“Kanaka Maoli*” OR<br>Maori* OR<br>Maaori* OR<br>Aborigin* OR<br>“Torres Strait Islander*” OR<br>Sami* OR<br>Saami* OR<br>(MH "Aboriginal Canadians+") OR<br>(MH "Arctic Peoples+") OR<br>(MH "First Nations of Australia+") OR<br>(MH "Maori") OR<br>(MH "Native Americans+")<br>)<br><br>OR<br><br>(<br>(Native* OR<br>Indigenous* OR | American Indian*.mp OR<br>Native American*.mp OR<br>First Nation OR<br>First Nations OR<br>Inuit*.mp OR<br>Greenlandic.mp OR<br>Metis*.mp OR<br>Two spirit*.mp OR<br>Two-spirit*.mp OR<br>Kanaka Maoli*.mp OR<br>Maori*.mp OR<br>Maaori*.mp OR<br>Aborigin* OR<br>Torres Strait Islander*.mp OR<br>Sami*.mp OR<br>Saami*.mp OR<br>exp eskimo-aleut people/ OR<br>alaska native/ OR<br>american indian/ OR<br>canadian aboriginal/ OR<br>first nation/ OR<br>exp Na-Dene people/ OR<br>"hopi (people)"/ OR<br>"pima (people)"/ OR<br>tohono o'odham/ OR<br>exp algonkian people/ OR<br>exp caddoan people/ OR<br>exp "iroquois (people)"/ OR<br>exp penutian people/ OR<br>exp "sioux (people)"/ OR | TITLE-ABS-KEY(<br>“American Indian*” OR<br>“Native American*” OR<br>“First Nation” OR<br>“First Nations” OR<br>Inuit* OR<br>Greenlandic OR<br>Metis* OR<br>“Two spirit*” OR<br>Two-spirit* OR<br>“Kanaka Maoli*” OR<br>Maori* OR<br>Maaori* OR<br>Aborigin* OR<br>“Torres Strait Islander*” OR<br>Sami* OR<br>Saami*)<br><br>OR<br><br>(<br>TITLE-ABS-KEY(<br>Native* OR<br>Indigenous*)<br><br>AND<br><br>TITLE-ABS-KEY(<br>Australia* OR<br>“Torres Strait*” OR | “American Indian” OR<br>“American Indians” OR<br>“Native American” OR<br>“Native Americans” OR<br>“First Nation” OR<br>“First Nations” OR<br>Inuit* OR<br>Greenlandic OR<br>Metis* OR<br>“Two spirit” OR<br>Two-spirit* OR<br>“Kanaka Maoli” OR<br>Maori* OR<br>Maaori* OR<br>Aborigin* OR<br>“Torres Strait Islander” OR<br>“Torres Strait Islanders” OR<br>Sami* OR<br>Saami*<br><br>OR<br><br>(<br>(Native* OR<br>Indigenous*)<br><br>AND<br><br>(Australia* OR<br>“Torres Strait” OR |

| | | | | | |
|---|---|---|---|---|---|
| | Indigenous peoples [MH])<br><br>AND<br><br>(Australia* [tiab] OR<br>Australia [MH] OR<br>Torres Strait* [tiab] OR<br>New South Wales* [tiab] OR<br>Queensland* [tiab] OR<br>South Australia* [tiab] OR<br>Tasmania* [tiab] OR<br>Victoria* [tiab] OR<br>Western Australia* [tiab] OR<br>Australian Capital Territor* [tiab] OR<br>Northern Territor* [tiab] OR<br>New Zealand* [tiab] OR<br>NZ [tiab] OR<br>Aotearoa* [tiab] OR<br>New Zealand [MH] OR<br>Norway* [tiab] OR<br>Norway [MH] OR<br>Sweden* [tiab] OR<br>Sweden [MH] OR<br>Arctic* [tiab] OR<br>Arctic Regions [MH] OR<br>North America* [tiab] OR<br>Greenland* [tiab] OR<br>Greenland [MH] OR<br>Canad* [tiab] OR<br>Canada [MH] OR<br>Ontario* [tiab] OR<br>Quebec* [tiab] OR<br>Nova Scotia* [tiab] OR<br>New Brunswick* [tiab] OR<br>Manitoba* [tiab] OR<br>British Columbia* [tiab] OR<br>Prince Edward Island* [tiab] OR | (MH "Indigenous Peoples+"))<br><br>AND<br><br>(Australia* OR<br>(MH "Australia+") OR<br>“Torres Strait*” OR<br>“New South Wales*” OR<br>Queensland* OR<br>“South Australia*” OR<br>Tasmania* OR<br>Victoria* OR<br>“Western Australia*” OR<br>“Australian Capital Territor*” OR<br>“Northern Territor*” OR<br>“New Zealand*” OR<br>NZ OR<br>Aotearoa* OR<br>(MH "New Zealand") OR<br>Norway* OR<br>(MH "Norway") OR<br>Sweden* OR<br>(MH "Sweden") OR<br>Arctic* OR<br>(MH "Arctic Regions") OR<br>“North America*” OR<br>Greenland* OR<br>(MH "Greenland") OR<br>Canad* OR<br>(MH "Canada+") OR<br>Ontario* OR<br>Quebec* OR<br>“Nova Scotia*” OR<br>“New Brunswick*” OR<br>Manitoba* OR<br>“British Columbia*” OR<br>“Prince Edward Island*” OR | metis/ OR<br>"sami (people)"/ OR<br>indigenous australian/ OR<br>exp australian aborigine/ OR<br>torres strait islander/ OR<br>oceanic ancestry group/ OR<br>native hawaiian/ OR<br>"maori (people)"/<br><br>OR<br><br>(<br>(Native* OR<br>Indigenous* OR<br>indigenous people/)<br><br>AND<br><br>(Australia*.mp OR<br>exp Australia/ OR<br>Torres Strait*.mp OR<br>New South Wales*.mp OR<br>Queensland*.mp OR<br>South Australia*.mp OR<br>Tasmania*.mp OR<br>Victoria*.mp OR<br>Western Australia*.mp OR<br>Australian Capital Territor*.mp OR<br>Northern Territor*.mp OR<br>New Zealand*.mp OR<br>NZ.mp OR<br>Aotearoa*.mp OR<br>New Zealand/ OR<br>Norway*.mp OR<br>exp Norway/ OR<br>Sweden*.mp OR<br>Sweden/ OR | “New South Wales*” OR<br>Queensland* OR<br>“South Australia*” OR<br>Tasmania* OR<br>Victoria* OR<br>“Western Australia*” OR<br>“Australian Capital Territor*” OR<br>“Northern Territor*” OR<br>“New Zealand*” OR<br>NZ OR<br>Aotearoa* OR<br>Norway* OR<br>Sweden* OR<br>Arctic* OR<br>“North America*” OR<br>Greenland* OR<br>Canad* OR<br>Ontario* OR<br>Quebec* OR<br>“Nova Scotia*” OR<br>“New Brunswick*” OR<br>Manitoba* OR<br>“British Columbia*” OR<br>“Prince Edward Island*” OR<br>Saskatchewan* OR<br>Alberta* OR<br>Newfoundland* OR<br>Labrador* OR<br>“Northwest Territories*” OR<br>Yukon* OR<br>Nunavut* OR<br>“United States*” OR<br>USA OR<br>U.S.A* OR<br>Alabama* OR<br>Alaska* OR<br>Arizona* OR | “New South Wales” OR<br>Queensland* OR<br>“South Australia” OR<br>Tasmania* OR<br>Victoria* OR<br>“Western Australia” OR<br>“Australian Capital Territory” OR<br>“Northern Territory” OR<br>“New Zealand” OR<br>NZ OR<br>Aotearoa* OR<br>Norway* OR<br>Sweden* OR<br>Arctic* OR<br>“North America” OR<br>Greenland* OR<br>Canad* OR<br>Ontario* OR<br>Quebec* OR<br>“Nova Scotia” OR<br>“New Brunswick” OR<br>Manitoba* OR<br>“British Columbia” OR<br>“Prince Edward Island” OR<br>Saskatchewan* OR<br>Alberta* OR<br>Newfoundland* OR<br>Labrador* OR<br>“Northwest Territories” OR<br>Yukon* OR<br>Nunavut* OR<br>“United States” OR<br>USA OR<br>U.S.A* OR<br>Alabama* OR<br>Alaska* OR<br>Arizona* OR |

| | | | | | |
|---|---|---|---|---|---|
| | Saskatchewan* [tiab] OR<br>Alberta* [tiab] OR<br>Newfoundland* [tiab] OR<br>Labrador* [tiab] OR<br>Northwest Territories* [tiab] OR<br>Yukon* [tiab] OR<br>Nunavut* [tiab] OR<br>United States* [tiab] OR<br>USA [tiab] OR<br>U.S.A* [tiab] OR<br>United States [MH] OR<br>Alabama* [tiab] OR<br>Alaska* [tiab] OR<br>Arizona* [tiab] OR<br>Arkansas* [tiab] OR<br>California* [tiab] OR<br>Colorado* [tiab] OR<br>Connecticut* [tiab] OR<br>Delaware* [tiab] OR<br>Florida* [tiab] OR<br>Georgia* [tiab] OR<br>Hawai* [tiab] OR<br>Idaho* [tiab] OR<br>Illinois* [tiab] OR<br>Indiana* [tiab] OR<br>Iowa* [tiab] OR<br>Kansas* [tiab] OR<br>Kentucky* [tiab] OR<br>Louisiana* [tiab] OR<br>Maine* [tiab] OR<br>Maryland* [tiab] OR<br>Massachusetts* [tiab] OR<br>Michigan* [tiab] OR<br>Minnesota* [tiab] OR<br>Mississippi* [tiab] OR<br>Missouri* [tiab] OR<br>Montana* [tiab] OR<br>Nebraska* [tiab] OR | Saskatchewan* OR<br>Alberta* OR<br>Newfoundland* OR<br>Labrador* OR<br>“Northwest Territories*” OR<br>Yukon* OR<br>Nunavut* OR<br>“United States*” OR<br>USA OR<br>U.S.A* OR<br>(MH "United States+") OR<br>Alabama* OR<br>Alaska* OR<br>Arizona* OR<br>Arkansas* OR<br>California* OR<br>Colorado* OR<br>Connecticut* OR<br>Delaware* OR<br>Florida* OR<br>Georgia* OR<br>Hawai* OR<br>Idaho* OR<br>Illinois* OR<br>Indiana* OR<br>Iowa* OR<br>Kansas* OR<br>Kentucky* OR<br>Louisiana* OR<br>Maine* OR<br>Maryland* OR<br>Massachusetts* OR<br>Michigan* OR<br>Minnesota* OR<br>Mississippi* OR<br>Missouri* OR<br>Montana* OR<br>Nebraska* OR | Arctic*.mp OR<br>Arctic/ OR<br>North America*.mp OR<br>North America/ OR<br>Greenland*.mp OR<br>Greenland/ OR<br>Canad*.mp OR<br>exp Canada/ OR<br>Ontario*.mp OR<br>Quebec*.mp OR<br>Nova Scotia*.mp OR<br>New Brunswick*.mp OR<br>Manitoba*.mp OR<br>British Columbia*.mp OR<br>Prince Edward Island*.mp OR<br>Saskatchewan*.mp OR<br>Alberta*.mp OR<br>Newfoundland*.mp OR<br>Labrador*.mp OR<br>Northwest Territories*.mp OR<br>Yukon*.mp OR<br>Nunavut*.mp OR<br>United States*.mp OR<br>USA.mp OR<br>exp United States/ OR<br>Alabama*.mp OR<br>Alaska*.mp OR<br>Arizona*.mp OR<br>Arkansas*.mp OR<br>California*.mp OR<br>Colorado*.mp OR<br>Connecticut*.mp OR<br>Delaware*.mp OR<br>Florida*.mp OR<br>Georgia*.mp OR<br>Hawai*.mp OR<br>Idaho*.mp OR<br>Illinois*.mp OR | Arkansas* OR<br>California* OR<br>Colorado* OR<br>Connecticut* OR<br>Delaware* OR<br>Florida* OR<br>Georgia* OR<br>Hawai* OR<br>Idaho* OR<br>Illinois* OR<br>Indiana* OR<br>Iowa* OR<br>Kansas* OR<br>Kentucky* OR<br>Louisiana* OR<br>Maine* OR<br>Maryland* OR<br>Massachusetts* OR<br>Michigan* OR<br>Minnesota* OR<br>Mississippi* OR<br>Missouri* OR<br>Montana* OR<br>Nebraska* OR<br>Nevada* OR<br>“New Hampshire*” OR<br>“New Jersey*” OR<br>“New Mexico*” OR<br>“New York*” OR<br>“North Carolina*” OR<br>“North Dakota*” OR<br>Ohio* OR<br>Oklahoma* OR<br>Oregon* OR<br>Pennsylvania* OR<br>“Rhode Island*” OR<br>“South Carolina*” OR<br>“South Dakota*” OR | Arkansas* OR<br>California* OR<br>Colorado* OR<br>Connecticut* OR<br>Delaware* OR<br>Florida* OR<br>Georgia* OR<br>Hawai* OR<br>Idaho* OR<br>Illinois* OR<br>Indiana* OR<br>Iowa* OR<br>Kansas* OR<br>Kentucky* OR<br>Louisiana* OR<br>Maine* OR<br>Maryland* OR<br>Massachusetts* OR<br>Michigan* OR<br>Minnesota* OR<br>Mississippi* OR<br>Missouri* OR<br>Montana* OR<br>Nebraska* OR<br>Nevada* OR<br>“New Hampshire” OR<br>“New Jersey” OR<br>“New Mexico” OR<br>“New York” OR<br>“North Carolina” OR<br>“North Dakota” OR<br>Ohio* OR<br>Oklahoma* OR<br>Oregon* OR<br>Pennsylvania* OR<br>“Rhode Island” OR<br>“South Carolina” OR<br>“South Dakota” OR |

| | | | | | |
|---|---|---|---|---|---|
| | Nevada* [tiab] OR<br>New Hampshire* [tiab] OR<br>New Jersey* [tiab] OR<br>New Mexico* [tiab] OR<br>New York* [tiab] OR<br>North Carolina* [tiab] OR<br>North Dakota* [tiab] OR<br>Ohio* [tiab] OR<br>Oklahoma* [tiab] OR<br>Oregon* [tiab] OR<br>Pennsylvania* [tiab] OR<br>Rhode Island* [tiab] OR<br>South Carolina* [tiab] OR<br>South Dakota* [tiab] OR<br>Tennessee* [tiab] OR<br>Texas* [tiab] OR<br>Utah* [tiab] OR<br>Vermont* [tiab] OR<br>Virginia* [tiab] OR<br>Washington* [tiab] OR<br>West Virginia* [tiab] OR<br>Wisconsin* [tiab] OR<br>Wyoming* [tiab] OR<br>Washington D.C. [tiab] OR<br>District of Columbia [tiab])<br>) | Nevada* OR<br>“New Hampshire*” OR<br>“New Jersey*” OR<br>“New Mexico*” OR<br>“New York*” OR<br>“North Carolina*” OR<br>“North Dakota*” OR<br>Ohio* OR<br>Oklahoma* OR<br>Oregon* OR<br>Pennsylvania* OR<br>“Rhode Island*” OR<br>“South Carolina*” OR<br>“South Dakota*” OR<br>Tennessee* OR<br>Texas* OR<br>Utah* OR<br>Vermont* OR<br>Virginia* OR<br>Washington* OR<br>“West Virginia*” OR<br>Wisconsin* OR<br>Wyoming* OR<br>“Washington D.C.” OR<br>“District of Columbia”)<br>) | Indiana*.mp OR<br>Iowa*.mp OR<br>Kansas*.mp OR<br>Kentucky*.mp OR<br>Louisiana*.mp OR<br>Maine*.mp OR<br>Maryland*.mp OR<br>Massachusetts*.mp OR<br>Michigan*.mp OR<br>Minnesota*.mp OR<br>Mississippi*.mp OR<br>Missouri*.mp OR<br>Montana*.mp OR<br>Nebraska*.mp OR<br>Nevada*.mp OR<br>New Hampshire*.mp OR<br>New Jersey*.mp OR<br>New Mexico*.mp OR<br>New York*.mp OR<br>North Carolina*.mp OR<br>North Dakota*.mp OR<br>Ohio*.mp OR<br>Oklahoma*.mp OR<br>Oregon*.mp OR<br>Pennsylvania*.mp OR<br>Rhode Island*.mp OR<br>South Carolina*.mp OR<br>South Dakota*.mp OR<br>Tennessee*.mp OR<br>Texas*.mp OR<br>Utah*.mp OR<br>Vermont*.mp OR<br>Virginia*.mp OR<br>Washington*.mp OR<br>West Virginia*.mp OR<br>Wisconsin*.mp OR<br>Wyoming*.mp OR<br>District of Columbia.mp) | Tennessee* OR<br>Texas* OR<br>Utah* OR<br>Vermont* OR<br>Virginia* OR<br>Washington* OR<br>“West Virginia*” OR<br>Wisconsin* OR<br>Wyoming* OR<br>“Washington D.C.” OR<br>“District of Columbia”)<br>) | Tennessee* OR<br>Texas* OR<br>Utah* OR<br>Vermont* OR<br>Virginia* OR<br>Washington* OR<br>“West Virginia” OR<br>Wisconsin* OR<br>Wyoming* OR<br>“Washington D.C.” OR<br>“District of Columbia”)<br>) |

| | | | | | |
|---|---|---|---|---|---|
| | | | ) | | |
| **Adolescents:** | Adol* [tiab] OR<br>Young* [tiab] OR<br>Youth*[tiab] OR<br>Teen*[tiab] OR<br>Adolescent [MH] OR<br>Young Adult [MH] | Adol* OR<br>Young* OR<br>Youth* OR<br>Teen* OR<br>(MH "Adolescence+") OR<br>(MH "Pregnancy in Adolescence") OR<br>(MH "Young Adult") | Adol*.mp OR<br>Young*.mp OR<br>Youth*.mp OR<br>Teen*.mp OR<br>exp adolescent/ OR<br>young adult/ | TITLE-ABS-KEY(<br>Adol* OR<br>Young* OR<br>Youth* OR<br>Teen*) | Adol* OR<br>Young* OR<br>Youth* OR<br>Teen* |
| **Qualitative research:** | Interview* [tiab] OR<br>Experience* [tiab] OR<br>Qualitative [tiab] OR<br>Theme* [tiab] OR<br>Thematic [tiab] OR<br>Focus group* [tiab] OR<br>Participatory [tiab] OR<br>Qualitative Research [MH:NOEXP] | Interview* OR<br>Experience* OR<br>Qualitative OR<br>Theme* OR<br>Thematic OR<br>"Focus group*" OR<br>Participatory OR<br>(MH "Qualitative Studies+") | Interview*.mp OR<br>Experience*.mp OR<br>Qualitative.mp OR<br>Theme*.mp OR<br>Thematic.mp OR<br>Focus group*.mp OR<br>Participatory.mp OR<br>qualitative research/ OR<br>qualitative analysis/ | TITLE-ABS-KEY(<br>Interview* OR<br>Experience* OR<br>Qualitative OR<br>Theme* OR<br>Thematic OR<br>"Focus group*" OR<br>Participatory) | Interview* OR<br>Experience* OR<br>Qualitative OR<br>Theme* OR<br>Thematic OR<br>"Focus group" OR<br>"Focus groups" OR<br>Participatory |
