# Supplementary material for: Health and well-being needs of Indigenous adolescents: a protocol for a scoping review of qualitative studies
Source: BMJ Open. 2024 May 20;14(5):e079942. doi: 10.1136/bmjopen-2023-079942 (PMC11110593; doi:10.1136/bmjopen-2023-079942)
Supplement: Author's manuscript [file bmjopen-2023-079942.draft_revisions.pdf]

BMJ Open is committed to open peer review. As part of this commitment we make the peer review history of every article we publish publicly available.

When an article is published we post the peer reviewers' comments and the authors' responses online. We also post the versions of the paper that were used during peer review. These are the versions that the peer review comments apply to.

The versions of the paper that follow are the versions that were submitted during the peer review process. They are not the versions of record or the final published versions. They should not be cited or distributed as the published version of this manuscript.

BMJ Open is an open access journal and the full, final, typeset and author-corrected version of record of the manuscript is available on our site with no access controls, subscription charges or pay-per-view fees (http://bmjopen.bmj.com).

If you have any questions on BMJ Open's open peer review process please email info.bmjopen@bmj.com

BMJ Open

The health and wellbeing needs of Indigenous adolescents: A protocol for a scoping review of qualitative literature

Journal:	*BMJ Open*
Manuscript ID	bmjopen-2023-079942
Article Type:	Protocol
Date Submitted by the Author:	16-Sep-2023

Complete List of Authors:	Sise, Andrew; University of Otago, Ngāi Tahu Māori Health Research Unit<br>Azzopardi, Peter S; Telethon Kids Institute, Adolescent Health and Wellbeing; The University of Melbourne, Centre for Adolescent Health, MCRI, Department of Paediatrics<br>Brown, Alex; Australian National University, National Centre for Indigenous Genomics; Telethon Kids Institute<br>Tewhaiti-Smith, Jordan Michael; Medical Research Institute of New Zealand<br>Westhead, Seth; South Australian Health and Medical Research Institute, Wardliparingga Aboriginal Health Equity Theme<br>Kurji, Jaameeta; South Australian Health and Medical Research Institute, Wardliparingga Aboriginal Health Equity Theme; University of Ottawa Faculty of Medicine, School of Epidemiology & Public Health<br>McDonough, Daniel; South Australian Health and Medical Research Institute, Wardliparingga Aboriginal Health Equity Theme<br>Reilly, Rachel; South Australian Health and Medical Research Institute, Wardliparingga Aboriginal Health Equity Theme; The University of Adelaide School of Psychology<br>Bingham, Brittany; The University of British Columbia Faculty of Medicine, Social Medicine - Centre for Gender and Sexual Health Equity; Vancouver Coastal Health Authority, Indigenous Health<br>Brown, Ngiare; South Australian Health and Medical Research Institute, Wardliparingga Aboriginal Health Equity Theme<br>Cassidy-Mathews, Chenoa; The University of British Columbia School of Population and Public Health<br>Clark, Terryann; The University of Auckland Faculty of Medical and Health Sciences, School of Nursing<br>Elliott, Salenna; South Australian Health and Medical Research Institute, Wardliparingga Aboriginal Health Equity Theme<br>Finlay, Summer May; University of Wollongong School of Health and Society<br>Hansen, Ketil Lenert; UiT The Arctic University of Norway Faculty of Health Sciences, Regional Centre for Child, Youth Mental Health and Child Welfare North (RKBU North)<br>Harwood, Matire; University of Auckland, Department of General Practice and Primary Care<br>Knapp, Jonil; UiT The Arctic University of Norway Faculty of Health Sciences<br>Kvernmo, Siv; UiT The Arctic University of Norway, Department of Clinical Medicine<br>Lee, Crystal; University of New Mexico, College of Population Health

Watts, Ricky-Lee; None
Nadeau, Melanie; University of North Dakota School of Medicine and Health Sciences, Department of Indigenous Health
Pearson, Odette; South Australian Health and Medical Research Institute, Wardliparingga Aboriginal Health Equity Theme
Reading, J; Simon Fraser University Faculty of Health Sciences
Saewyc, Elizabeth; University of British Columbia School of Nursing
Seljenes, Amalie; UiT The Arctic University of Norway Faculty of Health Sciences
Stoor, Jon Petter; Umea University Department for Epidemiology and Global Health, Lávvuo-Research and Education for Sámi Health; UiT The Arctic University of Norway, Centre for Sami Health Research, Department of Community Medicine
Aubrey, Paula; University of North Dakota School of Medicine and Health Sciences, Indigenous Health Department
Crengle, Sue; University of Otago, Ngāi Tahu Māori Health Research Unit

Keywords: Adolescent, QUALITATIVE RESEARCH, Health Equity

SCHOLARONE™ Manuscripts

THE HEALTH AND WELLBEING NEEDS OF INDIGENOUS ADOLESCENTS: A PROTOCOL FOR A SCOPING REVIEW OF QUALITATIVE LITERATURE

Authors

Andrew Sise, Peter Azzopardi, Alex Brown, Jordan Tewhaiti-Smith, Seth Westhead, Jaameeta Kurji, Daniel McDonough, Rachel Reilly, Brittany Bingham, Ngiare Brown, Chenoa Cassidy-Matthews, Terryann C Clark, Salenna Elliott, Summer May Finlay, Ketil Lenert Hansen, Matire Harwood, Jonill Margrethe Fjellheim Knapp, Siv Kvernmo, Crystal Lee, Ricky Lee-Watts, Melanie Nadeau, Odette Pearson, Jeff Reading, Elizabeth Saewyc, Amalie Seljenes, Jon Petter Stoor, Paula Aubrey, Sue Crengle.

Corresponding Author

Prof Sue Crengle:

Email: sue.crengle@otago.ac.nz

Postal: Ngāi Tahu Māori Health Research Unit, PO Box 56, Dunedin 9054, New Zealand

WORD COUNT

2519 (including all headings; excluding abstract and references)

For peer review only

ABSTRACT

Introduction

Improving the health of Indigenous adolescents is central to addressing the health inequities faced by Indigenous peoples. To achieve this, it is critical to understand what is needed from the perspectives of Indigenous adolescents themselves. There have been many qualitative studies that capture the perspectives of Indigenous young people, but these have not been synthesized to date.

Methods and analysis

This scoping review will map qualitative studies conducted with Indigenous adolescents across Australia, Aotearoa New Zealand, Canada, the United States of America, Greenland, and Sami populations (Norway, and Sweden). We will systematically search databases (Pubmed, CINAHL, Embase, Scopus, the Informit Indigenous and Health Collections, Google Scholar, Arctic Health, the Circumpolar Health Bibliographic Database, Native Health Database, iPortal, and NZresearch.org) as well as specific websites and clearinghouses within each nation for qualitative studies that specifically sample and report data for Indigenous adolescents aged 10-24 years. We will limit our search to articles published in any language during the preceding five years given that needs have changed significantly over time. Two independent reviewers will identify relevant articles using a two-step process, with disagreements resolved by a third reviewer. Data will then be extracted from included articles using a standardised form, with descriptive synthesis focussing on key needs and priorities. This scoping review will be conducted and reported according to the Preferred Reporting Items for Systematic Reviews and Meta-Analyses extension for Scoping Reviews guidelines.

Ethics and Dissemination

Ethics approval is not required for this review. Findings will be disseminated via a peer reviewed journal article and will inform a broader project to develop evidence-based actions to improve Indigenous adolescent health.

KEYWORDS

Indigenous, adolescents, health and wellbeing, scoping review

For peer review only

STRENGTHS AND LIMITATIONS OF THIS STUDY

- A focus on qualitative research will allow us to identify the perspectives of young people.
- Synthesis of international literature will enable evaluation of both differences and commonalities in needs, between Indigenous youth living in different regions and contexts.
- This scoping review will identify knowledge gaps in this area, and set priorities for further primary qualitative enquiry.
- A limitation of this review is the potentially limited evidence for younger adolescents and for some included Indigenous peoples.
- A further limitation is that this review is restricted to Indigenous peoples in included high income countries.

For peer review only

BACKGROUND

Indigenous peoples, numbering nearly 500 million individuals worldwide,[1] inhabit diverse regions and possess a rich variety of cultures, languages, and traditions.[2]

Indigenous peoples have the right to enjoy the highest attainable standards of physical and mental health.[3] Yet globally, they experience health outcomes that are substantially poorer than those of non-Indigenous populations.[4] These differences, termed inequities due to their being both avoidable and unjust,[5] are manifestations of a broad range of challenges that have been faced by Indigenous peoples. Central to the development of these inequities has been the process of colonisation.[6] Since the first arrival of settlers, Indigenous peoples have been displaced from their lands, and experienced the destruction and degradation of natural environments. New infectious diseases, and harmful substances such as alcohol and tobacco have been introduced, often with devastating consequences. Long established traditions and practices have been suppressed and disrupted by unjust laws and regulations, and Indigenous peoples have been marginalised within dominant settler societies. Colonisation, harmful policies and racism are not confined to the past, continuing into the present day.[7] This has had, and continues to have, a profound impact on the health of Indigenous populations.[6]

Despite this Indigenous peoples have resisted, survived, and maintained their distinct identities as sovereign peoples.[2] Indigenous peoples also continue to hold unique knowledge and worldviews. Embracing and respecting these ways of knowing alongside Western paradigms of health and knowledge generation[8] will provide substantial opportunities for Indigenous health advancement. Although it is imperative that governments and institutions act to improve the health of Indigenous peoples, it is essential that Indigenous voices and perspectives are at the forefront of these efforts.[2]

In order to address inequities faced by Indigenous peoples, it will also be crucial to consider the health and wellbeing of Indigenous adolescents, who now represent a third of most Indigenous populations.[9] While adolescence has traditionally been considered the healthiest time in a person's life, we now understand it as a life phase where unique health needs emerge, particularly those related to sexual and reproductive health, mental health, substance use, and injury.[10] Adolescence is a pivotal time in physical, social, and emotional development, and a time when individuals develop their identity; this results in adolescents being particularly susceptible to racism and discrimination based on ethnicity, religion, disability, sexual orientation, gender expression, or other factors. It is a time when health risk behaviours prominently emerge, and are potentially

For peer review only

modifiable. Health and wellbeing during adolescence is an important determinant of intergenerational health.[11] Crucially, adolescents are also incredible agents of change, and bring unique skills and perspectives that can shape health and wellbeing for all.[12] As such, adolescence is increasingly recognised as a time at which investments in health and wellbeing can bring maximal benefits, both now and into the future.[10]

Despite its clear importance, global investment in adolescent health to date has been limited, at least in part due to a lack of data regarding adolescent health needs.[10] Currently, efforts such as the World Health Organisation's Global Action for Measurement of Adolescent Health (GAMA)[13] are underway, aiming to strengthen quantitative measurement of adolescent health needs. The success of these efforts, however, will remain dependent on the quality of data available at individual country level. In addition, Indigenous adolescents experience different health risks and experience substantial health inequities when compared to non-Indigenous adolescents, that may not be captured by existing data and indicator systems.[6, 9] To achieve the crucial task of improving health outcomes for Indigenous adolescent people, a specific focus on their health and wellbeing, which recognises their unique status as Indigenous peoples will also be required.

Presently, a global collective (the Global Collective for Indigenous Adolescent Health and Evidence-Based Action) is undertaking a project which seeks to enhance knowledge in this area and advocate for evidence-based action to improve Indigenous adolescent health.[12] This will involve both identifying relevant indicators through which Indigenous adolescent health can be monitored, and assembling the best available evidence to develop actions with which to respond. Initially, the collective will focus on Indigenous adolescents living within Australia, Aotearoa New Zealand, Alaska, Canada, the United States of America, Greenland, Norway, and Sweden.

As a part of this effort to measure and respond to Indigenous adolescent health, it is crucial to understand what is important for health and wellbeing from the perspectives of Indigenous adolescents themselves. Qualitative research is an ideal format for ascertainment of such information, because it allows researchers to hear from young people in their own voice and will enable young people to describe their own priorities, needs and solutions. A preliminary search has identified that there is an appreciable body of qualitative literature that is focussed on Indigenous adolescent health and wellbeing; this is also consistent with an earlier mapping of data available for Indigenous adolescents in Australia.[14] For example, qualitative studies have explored the health needs of Aboriginal and Torres Strait Islander adolescents living in rural settings in Australia,[15] perspectives of First Nations adolescent girls in Canada regarding the meaning of (and what is important for) a healthy body,[16] and Inuit youth perspectives on sexual health in Nunavut.[17]

For peer review only

However, a search of the Pubmed database has revealed no recent synthesis of this rich qualitative data. Scoping reviews are well suited to identifying key concepts within a body of literature, as well as mapping, reporting and discussing these concepts.[18]

This protocol is for a scoping review which aims to map the available qualitative literature for Indigenous adolescents in Australia, Aotearoa New Zealand, Canada, the United States of America, Greenland, Norway, and Sweden. We seek to map qualitative data that describes key issues and needs from the perspective of adolescents themselves.

METHODS

The development of this protocol was informed by the Joanna Briggs Institute (JBI) Scoping Review Methodology Guidelines.[19] This scoping review will be reported in accordance with the Preferred Reporting Items for Systematic Reviews and Meta-Analyses extension for Scoping Reviews (PRISMA-ScR) checklist.[20]

Eligibility criteria

Participants

Eligible studies will include participants who are Indigenous peoples of Aotearoa New Zealand, Alaska, Australia, Canada, the United States of America, Greenland, Norway, or Sweden, and are between 10 and 24 years of age.

As per United Nations practice,[2] a rigid definition of Indigenous status will not be used. Participants of a study will be considered Indigenous when this is clearly identified by the text of the associated article. Currently, the World Health Organisation defines adolescence as occurring between the ages of 10 and 19 years.[21] However, evolving understandings of the timing of biological maturation, and increasing delays in role transitions such as marriage and parenthood mean that it is now proposed that adolescence should be defined as occurring between the ages of 10 and 24 years.[22] The Global Collective has chosen the age range of 10 – 24 years to reflect this understanding. For this review, we will only include studies that specifically sample and report data for individuals ranging from 10 to 24 years of age.

Concept:

To be eligible, studies must report qualitative data from participants’ perspectives on their health and wellbeing.

For peer review only

Context:

To be eligible, a study must have been conducted within Aotearoa New Zealand, Australia, Canada, the United States of America, Greenland, Norway, or Sweden. Any context or setting within these nations or regions will be acceptable.

Publication type:

We will include peer-reviewed and grey literature; in Australia for example there are a number of high quality consultations with Aboriginal and Torres Strait Islander adolescents that are published by Government and non-Government organisations.[23] Mixed methods studies will be eligible only if the health needs and/or priorities which are presented were identified based on qualitative enquiry with Indigenous adolescent people.

Date and language limitations:

Publications in any language, published during the five calendar years prior to, and the year up until the search date will be eligible for inclusion. Evolving society, technology, and culture means that the determinants of adolescent health and wellbeing are likely to be changing over time. The window for study inclusion has been chosen to reflect this, as more contemporary studies will likely be of greater relevance. If insufficient articles are identified with publication dates within this period, this window may be extended to include earlier years.

Search strategy

This scoping review will follow a three part search strategy. Firstly, databases (including Google Scholar) will be systematically searched for relevant literature. Secondly, key journals, websites and clearinghouses relevant to Indigenous health in each Nation will be hand searched. Third and finally, the references of all included articles will be searched to identify any additional relevant articles that were not retrieved by the first two steps. Our search is also to be completed by a network of experts across each Nation who will bring knowledge of available resources and publications.

Databases

A systematic electronic search will be performed of Pubmed, CINAHL, Embase, Scopus, Informit (Indigenous and Health collections), Google Scholar, Arctic Health, the Circumpolar Health Bibliographic Database, Native Health Database (Canada and USA), iPortal (University of Saskatchewan), and NZresearch.org (Aotearoa New Zealand).

For peer review only

Search terms

The full search terms which will be applied to Pubmed, CINHAL, Embase, Scopus, and the Informit Indigenous and Health collections are provided in Supplementary File 1. These search terms were primarily developed in the Pubmed database using an iterative process consisting of repeated searches, analysis of included and excluded titles, and analysis of terms used in article keywords, titles and abstracts. Relevant Medical Subject Heading (MeSH) terms were identified using the National Library of Medicine's MeSH browser,[24] and search terms for identifying qualitative literature were adapted from terms presented in a 2017 analysis by Rogers *et al*.[25] Simple keyword (non-controlled-vocabulary) terms were translated from Pubmed to all other databases directly. For databases which used controlled vocabularies different to MeSH terms (CINAHL and Embase), searching of those vocabularies to identify equivalent or additional relevant vocabulary terms was also completed.

Selection of sources of evidence to include in the review

Once the searches have been conducted in all databases and duplicate citations removed, remaining citations will be screened using Covidence systematic review management software. Initially, titles and abstracts will be screened for potential relevance. The full texts of potentially relevant articles identified at the title/abstract screening stage will then be retrieved for assessment in detail against the inclusion criteria of the review. At both the title/abstract and full text stages, two independent reviewers will be used, with any disagreements resolved by a third reviewer. In the final reporting of the review, a flow diagram will be used to document the selection of articles for inclusion, and reasons for exclusion of articles at the full text stage will be documented in accordance with the PRISMA-ScR guidelines.[20]

Hand searching of specific websites, clearinghouses, journals and references:

Once the electronic search is complete, additional hand searching of specific websites, clearinghouses and journals relevant to Indigenous health, and the references of all studies identified as being eligible for the review, will be completed. Journals to be hand searched will include: *AlterNative*, the *Journal of Indigenous Wellbeing: Te Mauri Pimatisiwin*, *MAI Journal*, *ab-Original: Journal of Indigenous Studies and First Nations and First Peoples' Cultures,* the *International Journal of Indigenous Health*, *American Indian and Alaska Native Mental Health Research*, The *American Indian Culture and Research Journal*, the *Canadian Journal of Native Studies*, and the *International Journal of Circumpolar Health*.

For peer review only

Data extraction

Data extraction will be handled in two stages. First, for studies meeting inclusion criteria (Indigenous population AND adolescent focused AND qualitative in design) we will capture basic demographics of the study using a standardised form. This will include publication year, abbreviated reference, and whether the study focuses on understanding health and wellbeing, needs and priorities from the perspective of adolescents OR if the study focuses on a specific health issue/s (such as mental health). In the second phase, we will first look at those studies that explore broad issues, needs and priorities, such as that conducted by Mohajer and colleagues.[15] For these studies we will extract the aims and research questions, qualitative method(s), participants (including number, Indigenous people(s), age range, gender(s) represented), setting (including country/region), and the quality of the study from an Indigenous research perspective.[26] For issue-specific qualitative research, we will extract similar information but also map the specific issues which they focus on; we will use the priority needs framework for adolescents globally as developed by WHO as a guide for this mapping.[27] The relative focus of qualitative studies against this framework will also provide some insight into relative needs and priorities.

Analysis of evidence and presentation of results

Presentation of results will be descriptive, and efforts to assess the level of certainty of results (as might be done in a systematic review) will not be undertaken.[19]

Firstly, we will map the nature of the qualitative data overall for Indigenous adolescents. A descriptive table will be used to describe key characteristics of included studies including time of publication, age of participants, Indigenous peoples covered, and the focus of these qualitative studies. This will enable the available literature to be mapped and gaps in coverage to be identified.

We will then undertake separate analyses of the studies identified by this review, depending on the quality and nature of data identified. For example, we hope to be able to synthesize what the overall priorities and needs of Indigenous adolescents are from the descriptive data. We plan to use a simple descriptive analysis of qualitative content[19] to synthesize the health and wellbeing needs and priorities identified across included papers. We will code needs and priorities identified by individual studies (for example, stress, worry, poor sexual health, racism), then group these into higher level categories (health outcome, health risk, health determinant), using a process similar to that undertaken by Harfield and colleagues.[28] Alongside describing core health and wellbeing needs and priorities overall, these categories will enable (where data are sufficient) comparisons

For peer review only

across different demographic variables such as age group, country/region, and gender. A narrative description will be used to highlight the key findings of these analyses.

ETHICS AND DISSEMINATION

Ethical approval is not required for this scoping review. Dissemination will occur via two key avenues. Firstly, a report of the review and its findings will be published as a standalone peer reviewed journal article. Secondly, findings from this review will inform work currently underway by our Global Collective to identify key indicators for monitoring Indigenous adolescent health across nations, and synthesize evidence to develop appropriate responses.[12] The findings and recommendations of this wider project are intended to be published in an upcoming two-part series of journal articles.

CONCLUSION

Improving the health and wellbeing of Indigenous adolescents will be essential to addressing the inequities faced by Indigenous peoples.[9] With this scoping review, we will evaluate the peer reviewed qualitative literature pertaining to Indigenous adolescent perspectives on what is important for their health and wellbeing. In identifying what is important to adolescents themselves, this review will highlight areas for action. In addition, we will map the coverage of literature in this crucial area, and establish whether gaps exist that warrant further research. This will be a key step in the wider efforts of our global collective,[12] which aims to measure, respond to, and develop strategies to enhance Indigenous adolescent health.

For peer review only

ACKNOWLEDGEMENTS

The authors would like to thank Christy Ballard, Librarian at the University of Otago, for her input and review of the search strategy for this protocol.

AUTHOR'S CONTRIBUTIONS:

All authors conceived and developed the concept for this scoping review, as part of the Global Collective for Indigenous Adolescent Health and Evidence-Based Action. Andrew Sise, Salenna Elliott, Sue Crengle, Peter Azzopardi, Seth Westhead, Jordan Tewhaiti-Smith, Alex Brown, and Rachel Reilly developed the initial search protocol. All authors reviewed the search protocol critically and provided input from their respective nations. Andrew Sise produced initial drafts, then all authors undertook further drafting, critical review, and editing to produce the final manuscript. All authors have approved the final version for publication. Sue Crengle is responsible for the overall content as the guarantor.

FUNDING STATEMENT:

This work was supported by the Australian National Health and Medical Research Council (NHMRC) Centre for Research Excellence for Driving Investment in Global Adolescent Health (GNT 1171981). P Azzopardi is supported by a NHMRC Investigator grant (GNT 2008574). JP Stoor's participation was funded by FORTE: Swedish Research Council for Health, Working Life and Welfare (Grant number 2021-01337). J Tewhaiti-Smith and M Harwood's participation was supported by the Medical Research Institute of New Zealand. T Clark's participation was supported by Cure Kids.

COMPETING INTERESTS STATEMENT:

None declared.

PATIENT AND PUBLIC INVOLVEMENT:

There was no patient or public involvement in the development of this protocol.

For peer review only

REFERENCES

1. International Labour Organisation. Implementing the ILO Indigenous and Tribal Peoples Convention No 169: Towards an inclusive, sustainable and just future [Internet]. Geneva: International Labour Organisation 2019: Section 2.3. A large and diverse population; p. 49 - 58. Available from: https://www.ilo.org/global/publications/books/WCMS_735607/lang--en/index.htm
2. United Nations. State of the world's Indigenous peoples. New York: United Nations; 2009 [cited 09 Nov 2021]. Available from: https://www.un.org/esa/socdev/unpfii/documents/SOWIP/en/SOWIP_web.pdf
3. United Nations General Assembly. United Nations Declaration on the Rights of Indigenous Peoples. New York: United Nations; 2007 [cited 09 Nov 2021]. Available from: https://www.un.org/development/desa/indigenouspeoples/declaration-on-the-rights-of-indigenous-peoples.html
4. Anderson I, Robson B, Connolly M, et al. Indigenous and tribal peoples' health (The Lancet-Lowitja Institute Global Collaboration): a population study. *Lancet* 2016;388(10040):131-57. doi: https://www.doi.org/10.1016/s0140-6736(16)00345-7
5. World Health Organisation. Social Determinants of Health [Internet]. Geneva: World Health Organisation; [cited 10 Nov 2021]. Available from: https://www.who.int/health-topics/social-determinants-of-health#tab=tab_1.
6. Gracey M, King M. Indigenous health part 1: determinants and disease patterns. *Lancet* 2009;374(9683):65-75. doi: http://dx.doi.org/10.1016/S0140-6736(09)60914-4
7. Reid P, Robson B. Understanding health inequities. In: Robson B, Harris R. editors. Hauora: Māori standards of health IV A study of the years 2000 - 2005 [Internet]. Wellington: Te Rōpū Rangahau Hauora a Eru Pōmare; 2007 [cited 14 Oct 2021]: 1; p. 3 - 10. Available from: https://ndhadeliver.natlib.govt.nz/delivery/DeliveryManagerServlet?dps_pid=IE772060
8. Martin DH. Two-eyed seeing: a framework for understanding indigenous and non-indigenous approaches to indigenous health research. *Can J Nurs Res* 2012;44(2):20-43.
9. Azzopardi PS, Sawyer SM, Carlin JB, et al. Health and wellbeing of Indigenous adolescents in Australia: a systematic synthesis of population data. *Lancet* 2018;391(10122):766-82. doi: https://www.doi.org/10.1016/s0140-6736(17)32141-4
10. Patton GC, Sawyer SM, Santelli JS, et al. Our future: a Lancet commission on adolescent health and wellbeing. *Lancet* 2016;387(10036):2423-78. doi: https://doi.org/10.1016/S0140-6736(16)00579-1
11. Patton GC, Olsson CA, Skirbekk V, et al. Adolescence and the next generation. *Nature* 2018;554(7693):458-66. doi: https://doi.org/10.1038/nature25759
12. Brown ADH, Crengle S, Tewhaiti-Smith J, et al. The health and wellbeing of Indigenous adolescents: a global collective for an equitable and sustainable future. *Lancet* 2022;399(10322):341-43. doi: https://doi.org/10.1016/S0140-6736(21)02719-7
13. World Health Organisation. Global Action for Measurement of Adolescent Health (GAMA). Terms of Reference and Workplan - Phase 2 (August 2020 - February 2023). Geneva: World Health Organisation [cited 07 Dec 2021]. Available from: https://platform.who.int/data/maternal-newborn-child-adolescent-ageing/advisory-groups/gama
14. Azzopardi PS, Kennedy EC, Patton GC, et al. The quality of health research for young Indigenous Australians: systematic review. *Med J Aust* 2013;199(1):57-63. doi: https://doi.org/10.5694/mja12.11141
15. Mohajer N, Bessarab D, Earnest J. There should be more help out here! A qualitative study of the needs of Aboriginal adolescents in rural Australia. *Rural Remote Health* 2009;9(2):1137.

For peer review only

16. Shea JM, Poudrier J, Chad K, et al. Understanding the healthy body from the perspective of First Nations girls in the Battlefords Tribal Council region: A photovoice project. *Native Studies Review* 2011;20(1):27-57.
17. Healey G. Youth perspectives on sexually transmitted infections and sexual health in Northern Canada and implications for public health practice. *Int J Circumpolar Health* 2016;75(1):30706. doi: https://www.doi.org/10.3402/ijch.v75.30706
18. Munn Z, Peters MDJ, Stern C, et al. Systematic review or scoping review? Guidance for authors when choosing between a systematic or scoping review approach. *BMC Med Res Methodol* 2018;18(1):143. doi: https://www.doi.org/10.1186/s12874-018-0611-x
19. Peters M, Godfrey C, McInerney P, et al. Chapter 11: Scoping Reviews (2020 version). In: Aromataris E, Munn Z. editors. JBI Manual for Evidence Synthesis [Internet]. Adelaide: JBI 2020 [cited 17 Nov 2021]. Available from: https://synthesismanual.jbi.global
20. Tricco AC, Lillie E, Zarin W, et al. PRISMA extension for scoping reviews (PRISMA-ScR): checklist and explanation. *Ann Intern Med* 2018;169(7):467-73. doi: https://www.doi.org/10.7326/M18-0850
21. World Health Organisation. Adolescent health (overview) [Internet]. World Health Organisation; [cited 24 Nov 2021]. Available from: https://www.who.int/health-topics/adolescent-health#tab=tab_1.
22. Sawyer SM, Azzopardi PS, Wickremarathne D, et al. The age of adolescence. *Lancet Child Adolesc Health* 2018;2(3):223-28. doi: https://www.doi.org/10.1016/S2352-4642(18)30022-1
23. Azzopardi P, Pearson O, Brown N, et al. Suggested priority areas for Aboriginal and Torres Strait Islander adolescent health: A report to the Department of Health. 2019
24. National Library of Medicine. Medical Subject Headings Browser (accessed via Medical Subject Headings home page) [Internet]. Bethesda, Maryland, USA: National Library of Medicine; [updated 8 Sep 2021; cited 16 Mar 2022]. Available from: https://www.nlm.nih.gov/mesh/meshhome.html.
25. Rogers M, Bethel A, Abbott R. Locating qualitative studies in dementia on MEDLINE, EMBASE, CINAHL, and PsycINFO: a comparison of search strategies. *Res Syn Meth* 2018;9(4):579-86. doi: https://www.doi.org/10.1002/jrsm.1280
26. Harfield S, Pearson O, Morey K, et al. Assessing the quality of health research from an Indigenous perspective: the Aboriginal and Torres Strait Islander quality appraisal tool. *BMC Med Res Methodol* 2020;20(1):79. doi: https://doi.org/10.1186/s12874-020-00959-3
27. Guthold R, Moller A-B, Adebayo E, et al. Priority Areas for Adolescent Health Measurement. *J Adolesc Health* 2021;68(5):888-98. doi: https://doi.org/10.1016/j.jadohealth.2020.12.127
28. Harfield SG, Davy C, McArthur A, et al. Characteristics of Indigenous primary health care service delivery models: a systematic scoping review. *Global Health* 2018;14(1):12. doi: https://www.doi.org/10.1186/s12992-018-0332-2

For peer review only

SUPPLEMENTARY FILE 1: SEARCH TERMS

Table 1. Search terms for the Pubmed, CINAHL, Embase, Scopus and Informit databases. To use, copy the entire contents of each cell into the search box of the relevant database. After the contents of each box has been searched separately, combine queries using the AND operator.

**Search filters (combine with AND)**	**Terms (Pubmed)**	**Terms (CINAHL)**	**Terms (Embase)**	**Terms (Scopus)**	**Terms (Informit Indigenous and Health Collections)**
**Indigenous peoples of eligible geographical locations:**	(American Indian*[tiab] OR<br>Native American*[tiab] OR<br>First Nation [tiab] OR<br>First Nations [tiab] OR<br>Inuit*[tiab] OR<br>Greenlandic [tiab] OR<br>Metis*[tiab] OR<br>Two spirit* [tiab] OR<br>Two-spirit* [tiab] OR<br>Kanaka Maoli* [tiab] OR<br>Maori*[tiab] OR<br>Maaori*[tiab] OR<br>Aborigin*[tiab] OR<br>Torres Strait Islander*[tiab] OR<br>Sami*[tiab] OR<br>Saami* [tiab] OR<br>American Native Continental Ancestry Group [MH:noexp] OR<br>Indians, North American [MH] OR<br>Indigenous Canadians [MH] OR<br>Inuits [MH] OR<br>"Native Hawaiian or other Pacific Islander" [MH])<br><br>OR<br><br>(<br>(Native*[tiab] OR	("American Indian*" OR<br>"Native American*" OR<br>"First Nation" OR<br>"First Nations" OR<br>Inuit* OR<br>Greenlandic OR<br>Metis* OR<br>"Two spirit*" OR<br>Two-spirit* OR<br>"Kanaka Maoli*" OR<br>Maori* OR<br>Maaori* OR<br>Aborigin* OR<br>"Torres Strait Islander*" OR<br>Sami* OR<br>Saami* OR<br>(MH "Aboriginal Canadians+") OR<br>(MH "Arctic Peoples+") OR<br>(MH "First Nations of Australia+") OR<br>(MH "Maori") OR<br>(MH "Native Americans+")<br>)<br><br>OR<br><br>(<br>(Native* OR	American Indian*.mp OR<br>Native American*.mp OR<br>First Nation OR<br>First Nations OR<br>Inuit*.mp OR<br>Greenlandic.mp OR<br>Metis*.mp OR<br>Two spirit*.mp OR<br>Two-spirit*.mp OR<br>Kanaka Maoli*.mp OR<br>Maori*.mp OR<br>Maaori*.mp OR<br>Aborigin* OR<br>Torres Strait Islander*.mp OR<br>Sami*.mp OR<br>Saami*.mp OR<br>exp eskimo-aleut people/ OR<br>alaska native/ OR<br>american indian/ OR<br>canadian aboriginal/ OR<br>first nation/ OR<br>exp Na-Dene people/ OR<br>"hopi (people)"/ OR<br>"pima (people)"/ OR<br>tohono o'odham/ OR<br>exp algonkian people/ OR<br>exp caddoan people/ OR<br>exp "iroquois (people)"/ OR<br>exp penutian people/ OR	TITLE-ABS-KEY(<br>"American Indian*" OR<br>"Native American*" OR<br>"First Nation" OR<br>"First Nations" OR<br>Inuit* OR<br>Greenlandic OR<br>Metis* OR<br>"Two spirit*" OR<br>Two-spirit* OR<br>"Kanaka Maoli*" OR<br>Maori* OR<br>Maaori* OR<br>Aborigin* OR<br>"Torres Strait Islander*" OR<br>Sami* OR<br>Saami*)<br><br>OR<br><br>(<br>TITLE-ABS-KEY(<br>Native* OR<br>Indigenous*)<br><br>AND<br><br>TITLE-ABS-KEY(<br>Australia* OR	"American Indian" OR<br>"American Indians" OR<br>"Native American" OR<br>"Native Americans" OR<br>"First Nation" OR<br>"First Nations" OR<br>Inuit* OR<br>Greenlandic OR<br>Metis* OR<br>"Two spirit" OR<br>Two-spirit* OR<br>"Kanaka Maoli" OR<br>Maori* OR<br>Maaori* OR<br>Aborigin* OR<br>"Torres Strait Islander" OR<br>"Torres Strait Islanders" OR<br>Sami* OR<br>Saami*<br><br>OR<br><br>(<br>(Native* OR<br>Indigenous*)<br><br>AND<br><br>(Australia* OR

For peer review only

	Indigenous*[tiab] OR<br>Indigenous peoples [MH])<br><br>AND<br><br>(Australia* [tiab] OR<br>Australia [MH] OR<br>Torres Strait* [tiab] OR<br>New South Wales* [tiab] OR<br>Queensland* [tiab] OR<br>South Australia* [tiab] OR<br>Tasmania* [tiab] OR<br>Victoria* [tiab] OR<br>Western Australia* [tiab] OR<br>Australian Capital Territor* [tiab] OR<br>Northern Territor* [tiab] OR<br>New Zealand* [tiab] OR<br>NZ [tiab] OR<br>Aotearoa* [tiab] OR<br>New Zealand [MH] OR<br>Norway* [tiab] OR<br>Norway [MH] OR<br>Sweden* [tiab] OR<br>Sweden [MH] OR<br>Arctic* [tiab] OR<br>Arctic Regions [MH] OR<br>North America* [tiab] OR<br>Greenland* [tiab] OR<br>Greenland [MH] OR<br>Canad* [tiab] OR<br>Canada [MH] OR<br>Ontario* [tiab] OR<br>Quebec* [tiab] OR<br>Nova Scotia* [tiab] OR<br>New Brunswick* [tiab] OR<br>Manitoba* [tiab] OR<br>British Columbia* [tiab] OR	Indigenous* OR<br>(MH "Indigenous Peoples+"))<br><br>AND<br><br>(Australia* OR<br>(MH "Australia+") OR<br>"Torres Strait*" OR<br>"New South Wales*" OR<br>Queensland* OR<br>"South Australia*" OR<br>Tasmania* OR<br>Victoria* OR<br>"Western Australia*" OR<br>"Australian Capital Territor*" OR<br>"Northern Territor*" OR<br>"New Zealand*" OR<br>NZ OR<br>Aotearoa* OR<br>(MH "New Zealand") OR<br>Norway* OR<br>(MH "Norway") OR<br>Sweden* OR<br>(MH "Sweden") OR<br>Arctic* OR<br>(MH "Arctic Regions") OR<br>"North America*" OR<br>Greenland* OR<br>(MH "Greenland") OR<br>Canad* OR<br>(MH "Canada+") OR<br>Ontario* OR<br>Quebec* OR<br>"Nova Scotia*" OR<br>"New Brunswick*" OR<br>Manitoba* OR<br>"British Columbia*" OR	exp "sioux (people)"/ OR<br>metis/ OR<br>"sami (people)"/ OR<br>indigenous australian/ OR<br>exp australian aborigine/ OR<br>torres strait islander/ OR<br>oceanic ancestry group/ OR<br>native hawaiian/ OR<br>"maori (people)"/<br><br>OR<br><br>(<br>(Native* OR<br>Indigenous* OR<br>indigenous people/)<br><br>AND<br><br>(Australia*.mp OR<br>exp Australia/ OR<br>Torres Strait*.mp OR<br>New South Wales*.mp OR<br>Queensland*.mp OR<br>South Australia*.mp OR<br>Tasmania*.mp OR<br>Victoria*.mp OR<br>Western Australia*.mp OR<br>Australian Capital Territor*.mp OR<br>Northern Territor*.mp OR<br>New Zealand*.mp OR<br>NZ.mp OR<br>Aotearoa*.mp OR<br>New Zealand/ OR<br>Norway*.mp OR<br>exp Norway/ OR<br>Sweden*.mp OR	"Torres Strait*" OR<br>"New South Wales*" OR<br>Queensland* OR<br>"South Australia*" OR<br>Tasmania* OR<br>Victoria* OR<br>"Western Australia*" OR<br>"Australian Capital Territor*" OR<br>"Northern Territor*" OR<br>"New Zealand*" OR<br>NZ OR<br>Aotearoa* OR<br>Norway* OR<br>Sweden* OR<br>Arctic* OR<br>"North America*" OR<br>Greenland* OR<br>Canad* OR<br>Ontario* OR<br>Quebec* OR<br>"Nova Scotia*" OR<br>"New Brunswick*" OR<br>Manitoba* OR<br>"British Columbia*" OR<br>"Prince Edward Island*" OR<br>Saskatchewan* OR<br>Alberta* OR<br>Newfoundland* OR<br>Labrador* OR<br>"Northwest Territories*" OR<br>Yukon* OR<br>Nunavut* OR<br>"United States*" OR<br>USA OR<br>U.S.A* OR<br>Alabama* OR<br>Alaska* OR	"Torres Strait" OR<br>"New South Wales" OR<br>Queensland* OR<br>"South Australia" OR<br>Tasmania* OR<br>Victoria* OR<br>"Western Australia" OR<br>"Australian Capital Territory" OR<br>"Northern Territory" OR<br>"New Zealand" OR<br>NZ OR<br>Aotearoa* OR<br>Norway* OR<br>Sweden* OR<br>Arctic* OR<br>"North America" OR<br>Greenland* OR<br>Canad* OR<br>Ontario* OR<br>Quebec* OR<br>"Nova Scotia" OR<br>"New Brunswick" OR<br>Manitoba* OR<br>"British Columbia" OR<br>"Prince Edward Island" OR<br>Saskatchewan* OR<br>Alberta* OR<br>Newfoundland* OR<br>Labrador* OR<br>"Northwest Territories" OR<br>Yukon* OR<br>Nunavut* OR<br>"United States" OR<br>USA OR<br>U.S.A* OR<br>Alabama* OR<br>Alaska* OR

For peer review only

	Prince Edward Island* [tiab] OR<br>Saskatchewan* [tiab] OR<br>Alberta* [tiab] OR<br>Newfoundland* [tiab] OR<br>Labrador* [tiab] OR<br>Northwest Territories* [tiab] OR<br>Yukon* [tiab] OR<br>Nunavut* [tiab] OR<br>United States* [tiab] OR<br>USA [tiab] OR<br>U.S.A* [tiab] OR<br>United States [MH] OR<br>Alabama* [tiab] OR<br>Alaska* [tiab] OR<br>Arizona* [tiab] OR<br>Arkansas* [tiab] OR<br>California* [tiab] OR<br>Colorado* [tiab] OR<br>Connecticut* [tiab] OR<br>Delaware* [tiab] OR<br>Florida* [tiab] OR<br>Georgia* [tiab] OR<br>Hawai* [tiab] OR<br>Idaho* [tiab] OR<br>Illinois* [tiab] OR<br>Indiana* [tiab] OR<br>Iowa* [tiab] OR<br>Kansas* [tiab] OR<br>Kentucky* [tiab] OR<br>Louisiana* [tiab] OR<br>Maine* [tiab] OR<br>Maryland* [tiab] OR<br>Massachusetts* [tiab] OR<br>Michigan* [tiab] OR<br>Minnesota* [tiab] OR<br>Mississippi* [tiab] OR<br>Missouri* [tiab] OR<br>Montana* [tiab] OR	"Prince Edward Island*" OR<br>Saskatchewan* OR<br>Alberta* OR<br>Newfoundland* OR<br>Labrador* OR<br>"Northwest Territories*" OR<br>Yukon* OR<br>Nunavut* OR<br>"United States*" OR<br>USA OR<br>U.S.A* OR<br>(MH "United States+") OR<br>Alabama* OR<br>Alaska* OR<br>Arizona* OR<br>Arkansas* OR<br>California* OR<br>Colorado* OR<br>Connecticut* OR<br>Delaware* OR<br>Florida* OR<br>Georgia* OR<br>Hawai* OR<br>Idaho* OR<br>Illinois* OR<br>Indiana* OR<br>Iowa* OR<br>Kansas* OR<br>Kentucky* OR<br>Louisiana* OR<br>Maine* OR<br>Maryland* OR<br>Massachusetts* OR<br>Michigan* OR<br>Minnesota* OR<br>Mississippi* OR<br>Missouri* OR<br>Montana* OR	Sweden/ OR<br>Arctic*.mp OR<br>Arctic/ OR<br>North America*.mp OR<br>North America/ OR<br>Greenland*.mp OR<br>Greenland/ OR<br>Canad*.mp OR<br>exp Canada/ OR<br>Ontario*.mp OR<br>Quebec*.mp OR<br>Nova Scotia*.mp OR<br>New Brunswick*.mp OR<br>Manitoba*.mp OR<br>British Columbia*.mp OR<br>Prince Edward Island*.mp OR<br>Saskatchewan*.mp OR<br>Alberta*.mp OR<br>Newfoundland*.mp OR<br>Labrador*.mp OR<br>Northwest Territories*.mp OR<br>Yukon*.mp OR<br>Nunavut*.mp OR<br>United States*.mp OR<br>USA.mp OR<br>exp United States/ OR<br>Alabama*.mp OR<br>Alaska*.mp OR<br>Arizona*.mp OR<br>Arkansas*.mp OR<br>California*.mp OR<br>Colorado*.mp OR<br>Connecticut*.mp OR<br>Delaware*.mp OR<br>Florida*.mp OR<br>Georgia*.mp OR<br>Hawai*.mp OR<br>Idaho*.mp OR	Arizona* OR<br>Arkansas* OR<br>California* OR<br>Colorado* OR<br>Connecticut* OR<br>Delaware* OR<br>Florida* OR<br>Georgia* OR<br>Hawai* OR<br>Idaho* OR<br>Illinois* OR<br>Indiana* OR<br>Iowa* OR<br>Kansas* OR<br>Kentucky* OR<br>Louisiana* OR<br>Maine* OR<br>Maryland* OR<br>Massachusetts* OR<br>Michigan* OR<br>Minnesota* OR<br>Mississippi* OR<br>Missouri* OR<br>Montana* OR<br>Nebraska* OR<br>Nevada* OR<br>"New Hampshire*" OR<br>"New Jersey*" OR<br>"New Mexico*" OR<br>"New York*" OR<br>"North Carolina*" OR<br>"North Dakota*" OR<br>Ohio* OR<br>Oklahoma* OR<br>Oregon* OR<br>Pennsylvania* OR<br>"Rhode Island*" OR<br>"South Carolina*" OR	Arizona* OR<br>Arkansas* OR<br>California* OR<br>Colorado* OR<br>Connecticut* OR<br>Delaware* OR<br>Florida* OR<br>Georgia* OR<br>Hawai* OR<br>Idaho* OR<br>Illinois* OR<br>Indiana* OR<br>Iowa* OR<br>Kansas* OR<br>Kentucky* OR<br>Louisiana* OR<br>Maine* OR<br>Maryland* OR<br>Massachusetts* OR<br>Michigan* OR<br>Minnesota* OR<br>Mississippi* OR<br>Missouri* OR<br>Montana* OR<br>Nebraska* OR<br>Nevada* OR<br>"New Hampshire" OR<br>"New Jersey" OR<br>"New Mexico" OR<br>"New York" OR<br>"North Carolina" OR<br>"North Dakota" OR<br>Ohio* OR<br>Oklahoma* OR<br>Oregon* OR<br>Pennsylvania* OR<br>"Rhode Island" OR<br>"South Carolina" OR

For peer review only

	Nebraska* [tiab] OR	Nebraska* OR	Illinois*.mp OR	“South Dakota*” OR	“South Dakota” OR
	Nevada* [tiab] OR	Nevada* OR	Indiana*.mp OR	Tennessee* OR	Tennessee* OR
	New Hampshire* [tiab] OR	“New Hampshire*” OR	Iowa*.mp OR	Texas* OR	Texas* OR
	New Jersey* [tiab] OR	“New Jersey*” OR	Kansas*.mp OR	Utah* OR	Utah* OR
	New Mexico* [tiab] OR	“New Mexico*” OR	Kentucky*.mp OR	Vermont* OR	Vermont* OR
	New York* [tiab] OR	“New York*” OR	Louisiana*.mp OR	Virginia* OR	Virginia* OR
	North Carolina* [tiab] OR	“North Carolina*” OR	Maine*.mp OR	Washington* OR	Washington* OR
	North Dakota* [tiab] OR	“North Dakota*” OR	Maryland*.mp OR	“West Virginia*” OR	“West Virginia” OR
	Ohio* [tiab] OR	Ohio* OR	Massachusetts*.mp OR	Wisconsin* OR	Wisconsin* OR
	Oklahoma* [tiab] OR	Oklahoma* OR	Michigan*.mp OR	Wyoming* OR	Wyoming* OR
	Oregon* [tiab] OR	Oregon* OR	Minnesota*.mp OR	“Washington D.C.” OR	“Washington D.C.” OR
	Pennsylvania* [tiab] OR	Pennsylvania* OR	Mississippi*.mp OR	“District of Columbia”)	“District of Columbia”)
	Rhode Island* [tiab] OR	“Rhode Island*” OR	Missouri*.mp OR	)	)
	South Carolina* [tiab] OR	“South Carolina*” OR	Montana*.mp OR		
	South Dakota* [tiab] OR	“South Dakota*” OR	Nebraska*.mp OR		
	Tennessee* [tiab] OR	Tennessee* OR	Nevada*.mp OR		
	Texas* [tiab] OR	Texas* OR	New Hampshire*.mp OR		
	Utah* [tiab] OR	Utah* OR	New Jersey*.mp OR		
	Vermont* [tiab] OR	Vermont* OR	New Mexico*.mp OR		
	Virginia* [tiab] OR	Virginia* OR	New York*.mp OR		
	Washington* [tiab] OR	Washington* OR	North Carolina*.mp OR		
	West Virginia* [tiab] OR	“West Virginia*” OR	North Dakota*.mp OR		
	Wisconsin* [tiab] OR	Wisconsin* OR	Ohio*.mp OR		
	Wyoming* [tiab] OR	Wyoming* OR	Oklahoma*.mp OR		
	Washington D.C. [tiab] OR	“Washington D.C.” OR	Oregon*.mp OR		
	District of Columbia [tiab])	“District of Columbia”)	Pennsylvania*.mp OR		
	)	)	Rhode Island*.mp OR		
			South Carolina*.mp OR		
			South Dakota*.mp OR		
			Tennessee*.mp OR		
			Texas*.mp OR		
			Utah*.mp OR		
			Vermont*.mp OR		
			Virginia*.mp OR		
			Washington*.mp OR		
			West Virginia*.mp OR		
			Wisconsin*.mp OR		
			Wyoming*.mp OR		

For peer review only

			District of Columbia.mp)<br>)		
**Adolescents:**	Adol* [tiab] OR<br>Young* [tiab] OR<br>Youth*[tiab] OR<br>Teen*[tiab] OR<br>Adolescent [MH] OR<br>Young Adult [MH]	Adol* OR<br>Young* OR<br>Youth* OR<br>Teen* OR<br>(MH "Adolescence+") OR<br>(MH "Pregnancy in Adolescence") OR<br>(MH "Young Adult")	Adol*.mp OR<br>Young*.mp OR<br>Youth*.mp OR<br>Teen*.mp OR<br>exp adolescent/ OR<br>young adult/	TITLE-ABS-KEY(<br>Adol* OR<br>Young* OR<br>Youth* OR<br>Teen*)	Adol* OR<br>Young* OR<br>Youth* OR<br>Teen*
**Qualitative research:**	Interview* [tiab] OR<br>Experience* [tiab] OR<br>Qualitative [tiab] OR<br>Theme* [tiab] OR<br>Thematic [tiab] OR<br>Focus group* [tiab] OR<br>Participatory [tiab] OR<br>Qualitative Research [MH:NOEXP]	Interview* OR<br>Experience* OR<br>Qualitative OR<br>Theme* OR<br>Thematic OR<br>"Focus group*" OR<br>Participatory OR<br>(MH "Qualitative Studies+")	Interview*.mp OR<br>Experience*.mp OR<br>Qualitative.mp OR<br>Theme*.mp OR<br>Thematic.mp OR<br>Focus group*.mp OR<br>Participatory.mp OR<br>qualitative research/ OR<br>qualitative analysis/	TITLE-ABS-KEY(<br>Interview* OR<br>Experience* OR<br>Qualitative OR<br>Theme* OR<br>Thematic OR<br>"Focus group*" OR<br>Participatory)	Interview* OR<br>Experience* OR<br>Qualitative OR<br>Theme* OR<br>Thematic OR<br>"Focus group" OR<br>"Focus groups" OR<br>Participatory

For peer review only

BMJ Open

The health and wellbeing needs of Indigenous adolescents: A protocol for a scoping review of qualitative studies

Journal:	*BMJ Open*
Manuscript ID	bmjopen-2023-079942.R1
Article Type:	Protocol
Date Submitted by the Author:	09-Apr-2024
Complete List of Authors:	Sise, Andrew; University of Otago, Ngāi Tahu Māori Health Research Unit<br>Azzopardi, Peter S; Telethon Kids Institute, Adolescent Health and Wellbeing; The University of Melbourne, Centre for Adolescent Health, MCRI, Department of Paediatrics<br>Brown, Alex; Australian National University, National Centre for Indigenous Genomics; Telethon Kids Institute<br>Tewhaiti-Smith, Jordan Michael; Medical Research Institute of New Zealand<br>Westhead, Seth; South Australian Health and Medical Research Institute, Wardliparingga Aboriginal Health Equity Theme<br>Kurji, Jaameeta; South Australian Health and Medical Research Institute, Wardliparingga Aboriginal Health Equity Theme; University of Ottawa Faculty of Medicine, School of Epidemiology & Public Health<br>McDonough, Daniel; South Australian Health and Medical Research Institute, Wardliparingga Aboriginal Health Equity Theme<br>Reilly, Rachel; South Australian Health and Medical Research Institute, Wardliparingga Aboriginal Health Equity Theme; The University of Adelaide School of Psychology<br>Bingham, Brittany; The University of British Columbia Faculty of Medicine, Social Medicine - Centre for Gender and Sexual Health Equity; Vancouver Coastal Health Authority, Indigenous Health<br>Brown, Ngiare; South Australian Health and Medical Research Institute, Wardliparingga Aboriginal Health Equity Theme<br>Cassidy-Mathews, Chenoa; The University of British Columbia School of Population and Public Health<br>Clark, Terryann; The University of Auckland Faculty of Medical and Health Sciences, School of Nursing<br>Elliott, Salenna; South Australian Health and Medical Research Institute, Wardliparingga Aboriginal Health Equity Theme<br>Finlay, Summer May; University of Wollongong School of Health and Society<br>Hansen, Ketil Lenert; UiT The Arctic University of Norway Faculty of Health Sciences, Regional Centre for Child, Youth Mental Health and Child Welfare North (RKBU North)<br>Harwood, Matire; University of Auckland, Department of General Practice and Primary Care<br>Knapp, Jonil; UiT The Arctic University of Norway Faculty of Health Sciences<br>Kvernmo, Siv; UiT The Arctic University of Norway, Department of Clinical Medicine<br>Lee, Crystal; University of New Mexico, College of Population Health

	Watts, Ricky-Lee; None<br>Nadeau, Melanie; University of North Dakota School of Medicine and Health Sciences, Department of Indigenous Health<br>Pearson, Odette; South Australian Health and Medical Research Institute, Wardliparingga Aboriginal Health Equity Theme<br>Reading, J; Simon Fraser University Faculty of Health Sciences<br>Saewyc, Elizabeth; University of British Columbia School of Nursing<br>Seljenes, Amalie; UiT The Arctic University of Norway Faculty of Health Sciences<br>Stoor, Jon Petter; Umea University Department for Epidemiology and Global Health, Lávvuo-Research and Education for Sámi Health; UiT The Arctic University of Norway, Centre for Sami Health Research, Department of Community Medicine<br>Aubrey, Paula; University of North Dakota School of Medicine and Health Sciences, Indigenous Health Department<br>Crengle, Sue; University of Otago, Ngāi Tahu Māori Health Research Unit

**Primary Subject Heading**:	Public health
Secondary Subject Heading:	Public health
Keywords:	Adolescent, QUALITATIVE RESEARCH, Health Equity

SCHOLARONE™ Manuscripts

THE HEALTH AND WELLBEING NEEDS OF INDIGENOUS ADOLESCENTS: A PROTOCOL FOR A SCOPING REVIEW OF QUALITATIVE STUDIES

Authors

Andrew Sise, Peter Azzopardi, Alex Brown, Jordan Tewhaiti-Smith, Seth Westhead, Jaameeta Kurji, Daniel McDonough, Rachel Reilly, Brittany Bingham, Ngiare Brown, Chenoa Cassidy-Matthews, Terryann C Clark, Salenna Elliott, Summer May Finlay, Ketil Lenert Hansen, Matire Harwood, Jonill Margrethe Fjellheim Knapp, Siv Kvernmo, Crystal Lee, Ricky-Lee Watts, Melanie Nadeau, Odette Pearson, Jeff Reading, Elizabeth Saewyc, Amalie Seljenes, Jon Petter Stoor, Paula Aubrey, Sue Crengle.

Corresponding Author

Prof Sue Crengle:

Email: sue.crengle@otago.ac.nz

Postal: Ngāi Tahu Māori Health Research Unit, PO Box 56, Dunedin 9054, New Zealand

WORD COUNT

3087 (including all headings; excluding abstract and references)

For peer review only

ABSTRACT

Introduction

Improving the health of Indigenous adolescents is central to addressing the health inequities faced by Indigenous peoples. To achieve this, it is critical to understand what is needed from the perspectives of Indigenous adolescents themselves. There have been many qualitative studies that capture the perspectives of Indigenous young people, but synthesis of these has been limited to date.

Methods and analysis

This scoping review seeks to understand the specific health needs and priorities of Indigenous adolescents aged 10 – 24 years captured via qualitative studies conducted across Australia, Aotearoa New Zealand, Canada, the United States of America, Greenland, and Sami populations (Norway, and Sweden). A team of Indigenous and non-Indigenous researchers from these nations will systematically search PubMed (including the MEDLINE, PubMed Central, and Bookshelf databases), CINAHL, Embase, Scopus, the Informit Indigenous and Health Collections, Google Scholar, Arctic Health, the Circumpolar Health Bibliographic Database, Native Health Database, iPortal, and NZresearch.org, as well as specific websites and clearinghouses within each nation for qualitative studies. We will limit our search to articles published in any language during the preceding five years given that needs may have changed significantly over time. Two independent reviewers will identify relevant articles using a two-step process, with disagreements resolved by a third reviewer and the wider research group. Data will then be extracted from included articles using a standardised form, with descriptive synthesis focussing on key needs and priorities. This scoping review will be conducted and reported according to the Preferred Reporting Items for Systematic Reviews and Meta-Analyses extension for Scoping Reviews guidelines.

Ethics and Dissemination

Ethics approval was not required for this review. Findings will be disseminated via a peer reviewed journal article and will inform a broader international collaboration for Indigenous adolescent health to develop evidence-based actions and solutions.

KEYWORDS

Indigenous, adolescents, health and wellbeing, scoping review

For peer review only - http://bmjopen.bmj.com/site/about/guidelines.xhtml

For peer review only

STRENGTHS AND LIMITATIONS OF THIS STUDY

- The planned scoping review will explore qualitative literature which identifies the perspectives of Indigenous adolescents.
- International literature will be synthesized to identify differences and commonalities in needs between Indigenous youth living in different regions and contexts.
- The Global Collective undertaking this work is led by Indigenous people, and includes Indigenous and non-Indigenous researchers and youth advocates from across included nations.
- A limitation of this review is the potentially limited evidence for younger adolescents and for some included Indigenous peoples.
- A further limitation is that this review is restricted to Indigenous peoples in included high income countries.

For peer review only

BACKGROUND

Indigenous peoples, numbering nearly 500 million individuals worldwide,[1] inhabit diverse regions and possess a rich variety of cultures, languages, and traditions.[2]

Indigenous peoples have the right to enjoy the highest attainable standards of physical and mental health.[3] Yet globally, they experience health outcomes that are substantially poorer than those of non-Indigenous populations.[4] These differences, termed inequities due to their being both avoidable and unjust,[5] are manifestations of a broad range of challenges that have been faced by Indigenous peoples. Central to the development of these inequities has been the process of colonisation.[6] Since the first arrival of settlers, Indigenous peoples have been displaced from their lands, and experienced the destruction and degradation of natural environments. New infectious diseases, and harmful substances such as alcohol and tobacco have been introduced, often with devastating consequences. Long established traditions and practices have been suppressed and disrupted by unjust laws and regulations, and Indigenous peoples have been marginalised within dominant settler societies. Colonisation, harmful policies and racism are not confined to the past, continuing into the present day.[7] This has had, and continues to have, a profound impact on the health of Indigenous populations.[6]

Despite this Indigenous peoples have resisted, survived, and maintained their distinct identities as sovereign peoples.[2] Indigenous peoples also continue to hold unique knowledge and worldviews. Embracing and respecting these ways of knowing alongside Western paradigms of health and knowledge generation[8] will provide substantial opportunities for Indigenous health advancement. Although it is imperative that governments and institutions act to improve the health of Indigenous peoples, it is essential that Indigenous voices and perspectives are at the forefront of these efforts.[2]

In order to address inequities faced by Indigenous peoples, it will also be crucial to consider the health and wellbeing of Indigenous adolescents, who now represent a third of most Indigenous populations.[9] While adolescence has traditionally been considered the healthiest time in a person's life, we now understand it as a life phase where unique health needs emerge, particularly those related to sexual and reproductive health, mental health, substance use, and injury.[10] Adolescence is a pivotal time in physical, social, and emotional development, and a time when individuals develop their identity; this results in adolescents being particularly susceptible to racism and discrimination based on ethnicity, religion, disability, sexual orientation, gender expression, or other factors. It is a time when health risk behaviours prominently emerge, and are potentially

For peer review only

modifiable. Health and wellbeing during adolescence is an important determinant of intergenerational health.[11] Crucially, adolescents are also incredible agents of change, and bring unique skills and perspectives that can shape health and wellbeing for all.[12] As such, adolescence is increasingly recognised as a time at which investments in health and wellbeing can bring maximal benefits, both now and into the future.[10]

Despite its clear importance, global investment in adolescent health to date has been limited, at least in part due to a lack of data regarding adolescent health needs.[10] Currently, efforts such as the World Health Organisation's Global Action for Measurement of Adolescent Health (GAMA)[13] are underway, aiming to strengthen quantitative measurement of adolescent health needs. The success of these efforts, however, will remain dependent on the quality of data available at individual country level. In addition, Indigenous adolescents experience different health risks and experience substantial health inequities when compared to non-Indigenous adolescents, that may not be captured by existing data and indicator systems.[6, 9] To achieve the crucial task of improving health outcomes for Indigenous adolescent people, a specific focus on their health and wellbeing, which recognises their unique status as Indigenous peoples will also be required.

Presently, the Global Collective for Indigenous Adolescent Health and Evidence-Based Action (the Global Collective) is undertaking a project which seeks to enhance knowledge in this area and advocate for evidence-based action to improve Indigenous adolescent health.[12] This will involve both identifying relevant indicators through which Indigenous adolescent health can be monitored, and assembling the best available evidence to develop actions with which to respond. Initially, the Global Collective will focus on Indigenous adolescents (defined for this purpose as being 10 – 24 years of age) living within Australia, Aotearoa New Zealand, Alaska, Canada, the United States of America, Greenland, Norway, and Sweden. The Global Collective is led by Indigenous people, and includes Indigenous and non-Indigenous researchers and youth advocates from across these nations.

As a part of this effort to measure and respond to Indigenous adolescent health, it is crucial to understand what is important for health and wellbeing from the perspectives of Indigenous adolescents themselves. Qualitative research is an ideal format for ascertainment of such information, because it allows researchers to hear from young people in their own voice and will enable young people to describe their own priorities, needs and solutions. A preliminary search has identified that there is an appreciable body of qualitative literature that is focussed on Indigenous adolescent health and wellbeing; this is also consistent with an earlier mapping of data available for Indigenous adolescents in Australia.[14] For example, qualitative studies have explored the health needs of Aboriginal and Torres Strait Islander adolescents living in rural settings in Australia,[15]

For peer review only - http://bmjopen.bmj.com/site/about/guidelines.xhtml

perspectives of First Nations adolescent girls in Canada regarding the meaning of (and what is important for) a healthy body,[16] and Inuit youth perspectives on sexual health in Nunavut.[17] Recently, substantial progress has been made in the synthesis of this evidence, with a systematic review of peer-reviewed literature having examined and compared key aspects of wellbeing for Indigenous young people aged 18 years or less in Canada, Australia, Aotearoa New Zealand and the United States of America.[18] To build on these efforts and set the foundation for the planned work of the Global Collective,[12] further exploration is necessary. In particular, it will be important to identify and map evidence across all nations included within the Global Collective's workplan, identify literature for youth across the Global Collective's broader age definition of adolescence, and explore the appreciable body of grey literature that may be available for some nations. Scoping reviews are well suited to exploring and identifying key concepts within a body of literature, as well as mapping, reporting and discussing these concepts.[19]

This protocol is for a scoping review which aims to map the available qualitative literature for Indigenous adolescents in Australia, Aotearoa New Zealand, Canada, the United States of America, Greenland, Norway, and Sweden. We seek to map qualitative data that describes key issues and needs from the perspective of adolescents themselves.

METHODS

The development of this protocol was informed by the Joanna Briggs Institute (JBI) Scoping Review Methodology Guidelines.[20] This scoping review will be reported in accordance with the Preferred Reporting Items for Systematic Reviews and Meta-Analyses extension for Scoping Reviews (PRISMA-ScR) checklist.[21] The scoping review which this protocol describes is underway. We ran the preliminary search in electronic databases in October 2023 and to date have completed a title and abstract screen based on eligibility criteria. Full texts are currently being retrieved for further assessment and we will also hand search grey literature. The review is being undertaken by researchers across the Global Collective, with work relevant to each nation being undertaken by researchers and experts of that nation. We anticipate the review will be completed in late 2024.

Eligibility criteria

Participants

Eligible studies will include participants who are Indigenous peoples of Aotearoa New Zealand, Alaska, Australia, Canada, the United States of America, Greenland, Norway, or Sweden, and are between 10 and 24 years of age.

For peer review only - http://bmjopen.bmj.com/site/about/guidelines.xhtml

As per United Nations practice,[2] a rigid definition of Indigenous status will not be used. Participants of a study will be considered Indigenous when this is clearly identified by the text of the associated article. While it is acknowledged that Indigenous peoples inhabit many regions of the world, the included regions/nations of Aotearoa New Zealand, Alaska, Australia, Canada, the United States of America, Greenland, Norway, and Sweden have been chosen for the initial work of the Global Collective due to similarities in their colonial histories, data systems, and policy contexts.[12] Currently, the World Health Organisation defines adolescence as occurring between the ages of 10 and 19 years.[22] However, evolving understandings of the timing of biological maturation, and increasing delays in role transitions such as marriage and parenthood mean that it is now proposed that adolescence should be defined as occurring between the ages of 10 and 24 years.[23] The Global Collective has chosen the age range of 10 – 24 years to reflect this understanding, and to capture the profound health-shaping changes which occur during this pivotal stage of development.[12] For this review, we will only include studies that specifically sample and report data for individuals ranging from 10 to 24 years of age.

Concept:

To be eligible, studies must report qualitative data from participants' perspectives on their health and wellbeing.

Context:

To be eligible, a study must have been conducted within Aotearoa New Zealand, Australia, Canada, the United States of America, Greenland, Norway, or Sweden. Any context or setting within these nations or regions will be acceptable.

Publication type:

We will include peer-reviewed and grey literature; in Australia for example there are a number of high quality consultations with Aboriginal and Torres Strait Islander adolescents that are published by Government and non-Government organisations.[24] To be eligible, studies must report data attained using qualitative methods (e.g.; interviews, focus groups, photovoice) where youth participants were able to identify and express their own views and perspectives. Quantitative studies including, for example, analyses of responses to surveys that used pre-set questions determined by researchers will not be included. Mixed methods studies (including both qualitative and quantitative components) will be eligible only if the health needs and/or priorities which are presented were identified based on qualitative enquiry with Indigenous adolescent people.

For peer review only - http://bmjopen.bmj.com/site/about/guidelines.xhtml

For peer review only

Date and language limitations:

Publications in any language, published during the five calendar years prior to, and the year up until the search date will be eligible for inclusion. Evolving society, technology, and culture means that the determinants of adolescent health and wellbeing are likely to be changing over time. The window for study inclusion has been chosen to reflect this, as more contemporary studies will likely be of greater relevance. If insufficient articles are identified with publication dates within this period, this window may be extended to include earlier years.

Search strategy

This scoping review will follow a three part search strategy. Firstly, databases (including Google Scholar) will be systematically searched for relevant literature. Secondly, key journals, websites and clearinghouses relevant to Indigenous health in each Nation will be hand searched. Third and finally, the references of all included articles will be searched to identify any additional relevant articles that were not retrieved by the first two steps. Our search is also to be completed by a network of experts across each Nation who will bring knowledge of available resources and publications.

Databases

A systematic electronic search will be performed of PubMed (including the MEDLINE, PubMed Central, and Bookshelf databases), CINAHL, Embase, Scopus, Informit (Indigenous and Health collections), Google Scholar, Arctic Health, the Circumpolar Health Bibliographic Database, Native Health Database (Canada and USA), iPortal (University of Saskatchewan), and NZresearch.org (Aotearoa New Zealand).

Search terms

The full search terms which will be applied to PubMed, CINHAL, Embase, Scopus, and the Informit Indigenous and Health collections are provided in Supplementary File 1. These search terms were primarily developed in the PubMed search engine using an iterative process consisting of repeated searches, analysis of included and excluded titles, and analysis of terms used in article keywords, titles and abstracts. Relevant Medical Subject Heading (MeSH) terms were identified using the National Library of Medicine's MeSH browser,[25] and search terms for identifying qualitative literature were adapted from terms presented in a 2017 analysis by Rogers *et al.*[26] Simple keyword (non-controlled-vocabulary) terms were translated from the PubMed search string to all other databases directly. For databases which used controlled vocabularies different to MeSH terms

For peer review only - http://bmjopen.bmj.com/site/about/guidelines.xhtml

(CINAHL and Embase), searching of those vocabularies to identify equivalent or additional relevant vocabulary terms was also completed.

Selection of sources of evidence to include in the review

Once the searches have been conducted in all databases and duplicate citations removed, remaining citations will be screened using Covidence systematic review management software. Initially, titles and abstracts will be screened for potential relevance. The full texts of potentially relevant articles identified at the title/abstract screening stage will then be retrieved for assessment in detail against the inclusion criteria of the review. At both the title/abstract and full text stages, two independent reviewers will be used, with any disagreements resolved by a third reviewer. In the final reporting of the review, a flow diagram will be used to document the selection of articles for inclusion, and reasons for exclusion of articles at the full text stage will be documented in accordance with the PRISMA-ScR guidelines.[21]

Hand searching of specific websites, clearinghouses, journals and references:

Once the electronic search is complete, additional hand searching of specific websites, clearinghouses and journals relevant to Indigenous health, and the references of all studies identified as being eligible for the review, will be completed. Journals to be hand searched will include: *AlterNative*, the *Journal of Indigenous Wellbeing: Te Mauri Pimatisiwin*, *MAI Journal*, *ab-Original: Journal of Indigenous Studies and First Nations and First Peoples' Cultures,* the *International Journal of Indigenous Health*, *American Indian and Alaska Native Mental Health Research*, The *American Indian Culture and Research Journal*, the *Canadian Journal of Native Studies*, and the *International Journal of Circumpolar Health*.

Data extraction

Data extraction will be handled in two stages. First, for studies meeting inclusion criteria (Indigenous population AND adolescent focused AND qualitative in design) we will capture basic demographics of the study using a standardised form. This will include publication year, abbreviated reference, and whether the study focuses on understanding health and wellbeing, needs and priorities from the perspective of adolescents OR if the study focuses on a specific health issue/s (such as mental health). While this review is restricted to studies in which youth were able to identify and express their own views and perspectives, we will also consider the extent to which studies appear to reflect these perspectives and whether, for example, core findings appear based primarily on youth or the researcher’s recommendations. In the second phase, we will first look at those studies that explore

For peer review only - http://bmjopen.bmj.com/site/about/guidelines.xhtml

broad issues, needs and priorities, such as that conducted by Mohajer and colleagues.[15] For these studies we will extract the aims and research questions, qualitative method(s), participants (including number, Indigenous people(s), age range, gender(s) represented), setting (including country/region), and the quality of the study from an Indigenous research perspective.[27] For issue-specific qualitative research, we will extract similar information but also map the specific issues which they focus on; we will use the priority needs framework for adolescents globally as developed by WHO as a guide for this mapping.[28] The relative focus of qualitative studies against this framework will also provide some insight into relative needs and priorities.

Analysis of evidence and presentation of results

Analysis and presentation of results will be primarily descriptive, and efforts to assess the level of certainty of results or undertake more complex methods of synthesis such as meta-aggregation (as might be done in a qualitative systematic review) will not be undertaken.[20]

Firstly, we will map the nature of the qualitative data overall for Indigenous adolescents. A descriptive table will be used to describe key characteristics of included studies including time of publication, age of participants, Indigenous peoples covered, and the focus of these qualitative studies. This will enable the available literature to be mapped and gaps in coverage to be identified.

We will then undertake further analyses of the studies identified by this review. Due to the wide scope of included nations and data sources, these analyses will be inherently exploratory, and their final format will depend on the quality and nature of data identified. However, we hope to be able to synthesize the overall priorities and needs of Indigenous adolescents that have been identified from across the findings of our included studies. We plan to use a simple descriptive analysis of qualitative content[20] to synthesize the health and wellbeing needs and priorities which are identified from the findings of included literature. We will aim to code needs and priorities identified by individual studies (for example, stress, worry, poor sexual health, racism), then group these into higher level categories (e.g., health outcome, health risk, health determinant), using a process similar to that undertaken by Harfield and colleagues.[29] Alongside describing any core health and wellbeing needs and priorities that are apparent across the findings of many studies, these categories will enable (where data are sufficient) simple comparisons across different demographic variables such as age group, country/region, and gender. A narrative description will be used to highlight the key findings of these analyses.

Patient and public involvement:

There was no patient or public involvement in the development of this protocol.

For peer review only - http://bmjopen.bmj.com/site/about/guidelines.xhtml

For peer review only

ETHICS AND DISSEMINATION

Ethical approval is not required for this scoping review. Dissemination will occur via two key avenues. Firstly, a report of the review and its findings will be published as a standalone peer reviewed journal article. Secondly, findings from this review will inform work currently underway by our Global Collective to identify key indicators for monitoring Indigenous adolescent health across nations, and synthesize evidence to develop appropriate responses.[12] The findings and recommendations of this wider project are intended to be published in an upcoming two-part series of journal articles.

CONCLUSION

Improving the health and wellbeing of Indigenous adolescents will be essential to addressing the inequities faced by Indigenous peoples.[9] With this scoping review, we will evaluate the peer reviewed qualitative literature pertaining to Indigenous adolescent perspectives on what is important for their health and wellbeing. Having begun with a preliminary search in October 2023, this review is progressing with work relevant to each nation being led by researchers and experts of those nations. This will enable consideration of issues specific to each region, whilst also allowing the identification of common themes and development of overarching recommendations. By identifying what is important to adolescents themselves, this review will highlight areas for action. It will also map the coverage of literature in this crucial area, and establish whether gaps exist that warrant further research. Depending on the review’s findings this could include, for example, identifying priority areas for consultation and primary qualitative enquiry, or set the direction for further evidence syntheses to develop evidence-based responses. This will be a key step in the wider efforts of the Global Collective,[12] which aims to measure, respond to, and develop strategies to enhance Indigenous adolescent health.

For peer review only
For peer review only - http://bmjopen.bmj.com/site/about/guidelines.xhtml

ACKNOWLEDGEMENTS

The authors would like to thank Christy Ballard, Librarian at the University of Otago, for her input and review of the search strategy for this protocol.

AUTHOR'S CONTRIBUTIONS:

All authors (A Sise, P Azzopardi, AB, JT-S, SW, JK, DM, RR, BB, NB, CC-M, TCC, SE, SMF, KLH, MH, JMFK, SK, CL, R-LW, MN, OP, JR, ES, A Seljenes, JPS, P Aubrey, and SC) conceived and developed the concept for this scoping review collectively, as part of the Global Collective for Indigenous Adolescent Health and Evidence-Based Action. A Sise, SE, SC, P Azzopardi, SW, JT-S, AB, and RR developed the initial search protocol. P Azzopardi, AB, JT-S, SW, JK, DM, RR, BB, NB, CC-M, TCC, SE, SMF, KLH, MH, JMFK, SK, CL, R-LW, MN, OP, JR, ES, A Seljenes, JPS, P Aubrey, and SC reviewed the search protocol critically and provided input from their respective nations. A Sise produced initial manuscript drafts, then A Sise, P Azzopardi, AB, JT-S, SW, JK, DM, RR, BB, NB, CC-M, TCC, SE, SMF, KLH, MH, JMFK, SK, CL, R-LW, MN, OP, JR, ES, A Seljenes, JPS, P Aubrey, and SC undertook further drafting, critical review, and editing to produce the final manuscript, and approved the final manuscript for publication. SC is responsible for the overall content as the guarantor.

FUNDING STATEMENT:

This work was supported by the Australian National Health and Medical Research Council (NHMRC) Centre for Research Excellence for Driving Investment in Global Adolescent Health (GNT 1171981). P Azzopardi is supported by a NHMRC Investigator grant (GNT 2008574). JP Stoor's participation was funded by FORTE: Swedish Research Council for Health, Working Life and Welfare (grant number 2021-01337). S Kvernmo's participation was supported by funding from the Sámi National Centre for Mental Health and Substance Use (grant number S3-2020), and the Sámi Parliament of Norway (grant number RD006/20). J Tewhaiti-Smith and M Harwood's participation was supported by the Medical Research Institute of New Zealand (grant numbers not applicable). T Clark's participation was supported by a Cure Kids Professorial Chair (grant number not applicable). Funders have not been involved in the development of this protocol.

For peer review only - http://bmjopen.bmj.com/site/about/guidelines.xhtml

COMPETING INTERESTS STATEMENT:

None declared.

For peer review only

For peer review only - http://bmjopen.bmj.com/site/about/guidelines.xhtml

REFERENCES

1. International Labour Organisation. Implementing the ILO Indigenous and Tribal Peoples Convention No 169: Towards an inclusive, sustainable and just future [Internet]. Geneva: International Labour Organisation 2019: Section 2.3. A large and diverse population; p. 49 - 58. Available from: https://www.ilo.org/global/publications/books/WCMS_735607/lang--en/index.htm
2. United Nations. State of the world's Indigenous peoples. New York: United Nations; 2009 [cited 09 Nov 2021]. Available from: https://www.un.org/esa/socdev/unpfii/documents/SOWIP/en/SOWIP_web.pdf
3. United Nations General Assembly. United Nations Declaration on the Rights of Indigenous Peoples. New York: United Nations; 2007 [cited 09 Nov 2021]. Available from: https://www.un.org/development/desa/indigenouspeoples/declaration-on-the-rights-of-indigenous-peoples.html
4. Anderson I, Robson B, Connolly M, et al. Indigenous and tribal peoples' health (The Lancet-Lowitja Institute Global Collaboration): a population study. *Lancet* 2016;388(10040):131-57. doi: https://www.doi.org/10.1016/s0140-6736(16)00345-7
5. World Health Organisation. Social Determinants of Health [Internet]. Geneva: World Health Organisation; [cited 10 Nov 2021]. Available from: https://www.who.int/health-topics/social-determinants-of-health#tab=tab_1.
6. Gracey M, King M. Indigenous health part 1: determinants and disease patterns. *Lancet* 2009;374(9683):65-75. doi: http://dx.doi.org/10.1016/S0140-6736(09)60914-4
7. Reid P, Robson B. Understanding health inequities. In: Robson B, Harris R. editors. Hauora: Māori standards of health IV A study of the years 2000 - 2005 [Internet]. Wellington: Te Rōpū Rangahau Hauora a Eru Pōmare; 2007 [cited 14 Oct 2021]: 1; p. 3 - 10. Available from: https://ndhadeliver.natlib.govt.nz/delivery/DeliveryManagerServlet?dps_pid=IE772060
8. Martin DH. Two-eyed seeing: a framework for understanding indigenous and non-indigenous approaches to indigenous health research. *Can J Nurs Res* 2012;44(2):20-43.
9. Azzopardi PS, Sawyer SM, Carlin JB, et al. Health and wellbeing of Indigenous adolescents in Australia: a systematic synthesis of population data. *Lancet* 2018;391(10122):766-82. doi: https://www.doi.org/10.1016/s0140-6736(17)32141-4
10. Patton GC, Sawyer SM, Santelli JS, et al. Our future: a Lancet commission on adolescent health and wellbeing. *Lancet* 2016;387(10036):2423-78. doi: https://doi.org/10.1016/S0140-6736(16)00579-1
11. Patton GC, Olsson CA, Skirbekk V, et al. Adolescence and the next generation. *Nature* 2018;554(7693):458-66. doi: https://doi.org/10.1038/nature25759
12. Brown ADH, Crengle S, Tewhaiti-Smith J, et al. The health and wellbeing of Indigenous adolescents: a global collective for an equitable and sustainable future. *Lancet* 2022;399(10322):341-43. doi: https://doi.org/10.1016/S0140-6736(21)02719-7
13. World Health Organisation. Global Action for Measurement of Adolescent Health (GAMA). Terms of Reference and Workplan - Phase 2 (August 2020 - February 2023). Geneva: World Health Organisation [cited 04 Mar 2024]. Available from: https://www.who.int/groups/the-global-action-for-measurement-of-adolescent-health/about-us
14. Azzopardi PS, Kennedy EC, Patton GC, et al. The quality of health research for young Indigenous Australians: systematic review. *Med J Aust* 2013;199(1):57-63. doi: https://doi.org/10.5694/mja12.11141
15. Mohajer N, Bessarab D, Earnest J. There should be more help out here! A qualitative study of the needs of Aboriginal adolescents in rural Australia. *Rural Remote Health* 2009;9(2):1137.

For peer review only - http://bmjopen.bmj.com/site/about/guidelines.xhtml

16. Shea JM, Poudrier J, Chad K, et al. Understanding the healthy body from the perspective of First Nations girls in the Battlefords Tribal Council region: A photovoice project. *Native Studies Review* 2011;20(1):27-57.
17. Healey G. Youth perspectives on sexually transmitted infections and sexual health in Northern Canada and implications for public health practice. *Int J Circumpolar Health* 2016;75(1):30706. doi: https://www.doi.org/10.3402/ijch.v75.30706
18. Anderson K, Elder-Robinson E, Gall A, et al. Aspects of wellbeing for Indigenous youth in CANZUS countries: a systematic review. *Int J Environ Res Public Health* 2022;19:13688. doi: https://doi.org/10.3390/ijerph192013688
19. Munn Z, Peters MDJ, Stern C, et al. Systematic review or scoping review? Guidance for authors when choosing between a systematic or scoping review approach. *BMC Med Res Methodol* 2018;18(1):143. doi: https://www.doi.org/10.1186/s12874-018-0611-x
20. Peters M, Godfrey C, McInerney P, et al. Chapter 11: Scoping Reviews (2020 version). In: Aromataris E, Munn Z. editors. JBI Manual for Evidence Synthesis [Internet]. Adelaide: JBI 2020 [cited 17 Nov 2021]. Available from: https://synthesismanual.jbi.global
21. Tricco AC, Lillie E, Zarin W, et al. PRISMA extension for scoping reviews (PRISMA-ScR): checklist and explanation. *Ann Intern Med* 2018;169(7):467-73. doi: https://www.doi.org/10.7326/M18-0850
22. World Health Organisation. Adolescent health (overview) [Internet]. World Health Organisation; [cited 24 Nov 2021]. Available from: https://www.who.int/health-topics/adolescent-health#tab=tab_1.
23. Sawyer SM, Azzopardi PS, Wickremarathne D, et al. The age of adolescence. *Lancet Child Adolesc Health* 2018;2(3):223-28. doi: https://www.doi.org/10.1016/S2352-4642(18)30022-1
24. Azzopardi P, Pearson O, Brown N, et al. Suggested priority areas for Aboriginal and Torres Strait Islander adolescent health: A report to the Department of Health. 2019
25. National Library of Medicine. Medical Subject Headings Browser (accessed via Medical Subject Headings home page) [Internet]. Bethesda, Maryland, USA: National Library of Medicine; [updated 8 Sep 2021; cited 16 Mar 2022]. Available from: https://www.nlm.nih.gov/mesh/meshhome.html.
26. Rogers M, Bethel A, Abbott R. Locating qualitative studies in dementia on MEDLINE, EMBASE, CINAHL, and PsycINFO: a comparison of search strategies. *Res Syn Meth* 2018;9(4):579-86. doi: https://www.doi.org/10.1002/jrsm.1280
27. Harfield S, Pearson O, Morey K, et al. Assessing the quality of health research from an Indigenous perspective: the Aboriginal and Torres Strait Islander quality appraisal tool. *BMC Med Res Methodol* 2020;20(1):79. doi: https://doi.org/10.1186/s12874-020-00959-3
28. Guthold R, Moller A-B, Adebayo E, et al. Priority Areas for Adolescent Health Measurement. *J Adolesc Health* 2021;68(5):888-98. doi: https://doi.org/10.1016/j.jadohealth.2020.12.127
29. Harfield SG, Davy C, McArthur A, et al. Characteristics of Indigenous primary health care service delivery models: a systematic scoping review. *Global Health* 2018;14(1):12. doi: https://www.doi.org/10.1186/s12992-018-0332-2

For peer review only - http://bmjopen.bmj.com/site/about/guidelines.xhtml

SUPPLEMENTARY FILE 1: SEARCH TERMS

Table 1. Search terms for the Pubmed, CINAHL, Embase, Scopus and Informit databases. To use, copy the entire contents of each cell into the search box of the relevant database. After the contents of each box has been searched separately, combine queries using the AND operator.

**Search filters (combine with AND)**	**Terms (Pubmed)**	**Terms (CINAHL)**	**Terms (Embase)**	**Terms (Scopus)**	**Terms (Informit Indigenous and Health Collections)**
**Indigenous peoples of eligible geographical locations:**	(American Indian*[tiab] OR<br>Native American*[tiab] OR<br>First Nation [tiab] OR<br>First Nations [tiab] OR<br>Inuit*[tiab] OR<br>Greenlandic [tiab] OR<br>Metis*[tiab] OR<br>Two spirit* [tiab] OR<br>Two-spirit* [tiab] OR<br>Kanaka Maoli* [tiab] OR<br>Maori*[tiab] OR<br>Maaori*[tiab] OR<br>Aborigin*[tiab] OR<br>Torres Strait Islander*[tiab] OR<br>Sami*[tiab] OR<br>Saami* [tiab] OR<br>American Native Continental Ancestry Group [MH:noexp] OR<br>Indians, North American [MH] OR<br>Indigenous Canadians [MH] OR<br>Inuits [MH] OR<br>"Native Hawaiian or other Pacific Islander" [MH])<br><br>OR<br><br>(<br>(Native*[tiab] OR<br>Indigenous*[tiab] OR	("American Indian*" OR<br>"Native American*" OR<br>"First Nation" OR<br>"First Nations" OR<br>Inuit* OR<br>Greenlandic OR<br>Metis* OR<br>"Two spirit*" OR<br>Two-spirit* OR<br>"Kanaka Maoli*" OR<br>Maori* OR<br>Maaori* OR<br>Aborigin* OR<br>"Torres Strait Islander*" OR<br>Sami* OR<br>Saami* OR<br>(MH "Aboriginal Canadians+") OR<br>(MH "Arctic Peoples+") OR<br>(MH "First Nations of Australia+") OR<br>(MH "Maori") OR<br>(MH "Native Americans+")<br>)<br><br>OR<br><br>(<br>(Native* OR<br>Indigenous* OR	American Indian*.mp OR<br>Native American*.mp OR<br>First Nation OR<br>First Nations OR<br>Inuit*.mp OR<br>Greenlandic.mp OR<br>Metis*.mp OR<br>Two spirit*.mp OR<br>Two-spirit*.mp OR<br>Kanaka Maoli*.mp OR<br>Maori*.mp OR<br>Maaori*.mp OR<br>Aborigin* OR<br>Torres Strait Islander*.mp OR<br>Sami*.mp OR<br>Saami*.mp OR<br>exp eskimo-aleut people/ OR<br>alaska native/ OR<br>american indian/ OR<br>canadian aboriginal/ OR<br>first nation/ OR<br>exp Na-Dene people/ OR<br>"hopi (people)"/ OR<br>"pima (people)"/ OR<br>tohono o'odham/ OR<br>exp algonkian people/ OR<br>exp caddoan people/ OR<br>exp "iroquois (people)"/ OR<br>exp penutian people/ OR<br>exp "sioux (people)"/ OR	TITLE-ABS-KEY(<br>"American Indian*" OR<br>"Native American*" OR<br>"First Nation" OR<br>"First Nations" OR<br>Inuit* OR<br>Greenlandic OR<br>Metis* OR<br>"Two spirit*" OR<br>Two-spirit* OR<br>"Kanaka Maoli*" OR<br>Maori* OR<br>Maaori* OR<br>Aborigin* OR<br>"Torres Strait Islander*" OR<br>Sami* OR<br>Saami*)<br><br>OR<br><br>(<br>TITLE-ABS-KEY(<br>Native* OR<br>Indigenous*)<br><br>AND<br><br>TITLE-ABS-KEY(<br>Australia* OR<br>"Torres Strait*" OR	"American Indian" OR<br>"American Indians" OR<br>"Native American" OR<br>"Native Americans" OR<br>"First Nation" OR<br>"First Nations" OR<br>Inuit* OR<br>Greenlandic OR<br>Metis* OR<br>"Two spirit" OR<br>Two-spirit* OR<br>"Kanaka Maoli" OR<br>Maori* OR<br>Maaori* OR<br>Aborigin* OR<br>"Torres Strait Islander" OR<br>"Torres Strait Islanders" OR<br>Sami* OR<br>Saami*<br><br>OR<br><br>(<br>(Native* OR<br>Indigenous*)<br><br>AND<br><br>(Australia* OR<br>"Torres Strait" OR

For peer review only

	Indigenous peoples [MH])<br><br>AND<br><br>(Australia* [tiab] OR<br>Australia [MH] OR<br>Torres Strait* [tiab] OR<br>New South Wales* [tiab] OR<br>Queensland* [tiab] OR<br>South Australia* [tiab] OR<br>Tasmania* [tiab] OR<br>Victoria* [tiab] OR<br>Western Australia* [tiab] OR<br>Australian Capital Territor* [tiab] OR<br>Northern Territor* [tiab] OR<br>New Zealand* [tiab] OR<br>NZ [tiab] OR<br>Aotearoa* [tiab] OR<br>New Zealand [MH] OR<br>Norway* [tiab] OR<br>Norway [MH] OR<br>Sweden* [tiab] OR<br>Sweden [MH] OR<br>Arctic* [tiab] OR<br>Arctic Regions [MH] OR<br>North America* [tiab] OR<br>Greenland* [tiab] OR<br>Greenland [MH] OR<br>Canad* [tiab] OR<br>Canada [MH] OR<br>Ontario* [tiab] OR<br>Quebec* [tiab] OR<br>Nova Scotia* [tiab] OR<br>New Brunswick* [tiab] OR<br>Manitoba* [tiab] OR<br>British Columbia* [tiab] OR<br>Prince Edward Island* [tiab] OR	(MH "Indigenous Peoples+"))<br><br>AND<br><br>(Australia* OR<br>(MH "Australia+") OR<br>“Torres Strait*” OR<br>“New South Wales*” OR<br>Queensland* OR<br>“South Australia*” OR<br>Tasmania* OR<br>Victoria* OR<br>“Western Australia*” OR<br>“Australian Capital Territor*” OR<br>“Northern Territor*” OR<br>“New Zealand*” OR<br>NZ OR<br>Aotearoa* OR<br>(MH "New Zealand") OR<br>Norway* OR<br>(MH "Norway") OR<br>Sweden* OR<br>(MH "Sweden") OR<br>Arctic* OR<br>(MH "Arctic Regions") OR<br>“North America*” OR<br>Greenland* OR<br>(MH "Greenland") OR<br>Canad* OR<br>(MH "Canada+") OR<br>Ontario* OR<br>Quebec* OR<br>“Nova Scotia*” OR<br>“New Brunswick*” OR<br>Manitoba* OR<br>“British Columbia*” OR<br>“Prince Edward Island*” OR	metis/ OR<br>"sami (people)"/ OR<br>indigenous australian/ OR<br>exp australian aborigine/ OR<br>torres strait islander/ OR<br>oceanic ancestry group/ OR<br>native hawaiian/ OR<br>"maori (people)"/<br><br>OR<br><br>(<br>(Native* OR<br>Indigenous* OR<br>indigenous people/)<br><br>AND<br><br>(Australia*.mp OR<br>exp Australia/ OR<br>Torres Strait*.mp OR<br>New South Wales*.mp OR<br>Queensland*.mp OR<br>South Australia*.mp OR<br>Tasmania*.mp OR<br>Victoria*.mp OR<br>Western Australia*.mp OR<br>Australian Capital Territor*.mp OR<br>Northern Territor*.mp OR<br>New Zealand*.mp OR<br>NZ.mp OR<br>Aotearoa*.mp OR<br>New Zealand/ OR<br>Norway*.mp OR<br>exp Norway/ OR<br>Sweden*.mp OR<br>Sweden/ OR	“New South Wales*” OR<br>Queensland* OR<br>“South Australia*” OR<br>Tasmania* OR<br>Victoria* OR<br>“Western Australia*” OR<br>“Australian Capital Territor*” OR<br>“Northern Territor*” OR<br>“New Zealand*” OR<br>NZ OR<br>Aotearoa* OR<br>Norway* OR<br>Sweden* OR<br>Arctic* OR<br>“North America*” OR<br>Greenland* OR<br>Canad* OR<br>Ontario* OR<br>Quebec* OR<br>“Nova Scotia*” OR<br>“New Brunswick*” OR<br>Manitoba* OR<br>“British Columbia*” OR<br>“Prince Edward Island*” OR<br>Saskatchewan* OR<br>Alberta* OR<br>Newfoundland* OR<br>Labrador* OR<br>“Northwest Territories*” OR<br>Yukon* OR<br>Nunavut* OR<br>“United States*” OR<br>USA OR<br>U.S.A* OR<br>Alabama* OR<br>Alaska* OR<br>Arizona* OR	“New South Wales” OR<br>Queensland* OR<br>“South Australia” OR<br>Tasmania* OR<br>Victoria* OR<br>“Western Australia” OR<br>“Australian Capital Territory” OR<br>“Northern Territory” OR<br>“New Zealand” OR<br>NZ OR<br>Aotearoa* OR<br>Norway* OR<br>Sweden* OR<br>Arctic* OR<br>“North America” OR<br>Greenland* OR<br>Canad* OR<br>Ontario* OR<br>Quebec* OR<br>“Nova Scotia” OR<br>“New Brunswick” OR<br>Manitoba* OR<br>“British Columbia” OR<br>“Prince Edward Island” OR<br>Saskatchewan* OR<br>Alberta* OR<br>Newfoundland* OR<br>Labrador* OR<br>“Northwest Territories” OR<br>Yukon* OR<br>Nunavut* OR<br>“United States” OR<br>USA OR<br>U.S.A* OR<br>Alabama* OR<br>Alaska* OR<br>Arizona* OR

	Saskatchewan* [tiab] OR<br>Alberta* [tiab] OR<br>Newfoundland* [tiab] OR<br>Labrador* [tiab] OR<br>Northwest Territories* [tiab] OR<br>Yukon* [tiab] OR<br>Nunavut* [tiab] OR<br>United States* [tiab] OR<br>USA [tiab] OR<br>U.S.A* [tiab] OR<br>United States [MH] OR<br>Alabama* [tiab] OR<br>Alaska* [tiab] OR<br>Arizona* [tiab] OR<br>Arkansas* [tiab] OR<br>California* [tiab] OR<br>Colorado* [tiab] OR<br>Connecticut* [tiab] OR<br>Delaware* [tiab] OR<br>Florida* [tiab] OR<br>Georgia* [tiab] OR<br>Hawai* [tiab] OR<br>Idaho* [tiab] OR<br>Illinois* [tiab] OR<br>Indiana* [tiab] OR<br>Iowa* [tiab] OR<br>Kansas* [tiab] OR<br>Kentucky* [tiab] OR<br>Louisiana* [tiab] OR<br>Maine* [tiab] OR<br>Maryland* [tiab] OR<br>Massachusetts* [tiab] OR<br>Michigan* [tiab] OR<br>Minnesota* [tiab] OR<br>Mississippi* [tiab] OR<br>Missouri* [tiab] OR<br>Montana* [tiab] OR<br>Nebraska* [tiab] OR	Saskatchewan* OR<br>Alberta* OR<br>Newfoundland* OR<br>Labrador* OR<br>"Northwest Territories*" OR<br>Yukon* OR<br>Nunavut* OR<br>"United States*" OR<br>USA OR<br>U.S.A* OR<br>(MH "United States+") OR<br>Alabama* OR<br>Alaska* OR<br>Arizona* OR<br>Arkansas* OR<br>California* OR<br>Colorado* OR<br>Connecticut* OR<br>Delaware* OR<br>Florida* OR<br>Georgia* OR<br>Hawai* OR<br>Idaho* OR<br>Illinois* OR<br>Indiana* OR<br>Iowa* OR<br>Kansas* OR<br>Kentucky* OR<br>Louisiana* OR<br>Maine* OR<br>Maryland* OR<br>Massachusetts* OR<br>Michigan* OR<br>Minnesota* OR<br>Mississippi* OR<br>Missouri* OR<br>Montana* OR<br>Nebraska* OR	Arctic*.mp OR<br>Arctic/ OR<br>North America*.mp OR<br>North America/ OR<br>Greenland*.mp OR<br>Greenland/ OR<br>Canad*.mp OR<br>exp Canada/ OR<br>Ontario*.mp OR<br>Quebec*.mp OR<br>Nova Scotia*.mp OR<br>New Brunswick*.mp OR<br>Manitoba*.mp OR<br>British Columbia*.mp OR<br>Prince Edward Island*.mp OR<br>Saskatchewan*.mp OR<br>Alberta*.mp OR<br>Newfoundland*.mp OR<br>Labrador*.mp OR<br>Northwest Territories*.mp OR<br>Yukon*.mp OR<br>Nunavut*.mp OR<br>United States*.mp OR<br>USA.mp OR<br>exp United States/ OR<br>Alabama*.mp OR<br>Alaska*.mp OR<br>Arizona*.mp OR<br>Arkansas*.mp OR<br>California*.mp OR<br>Colorado*.mp OR<br>Connecticut*.mp OR<br>Delaware*.mp OR<br>Florida*.mp OR<br>Georgia*.mp OR<br>Hawai*.mp OR<br>Idaho*.mp OR<br>Illinois*.mp OR	Arkansas* OR<br>California* OR<br>Colorado* OR<br>Connecticut* OR<br>Delaware* OR<br>Florida* OR<br>Georgia* OR<br>Hawai* OR<br>Idaho* OR<br>Illinois* OR<br>Indiana* OR<br>Iowa* OR<br>Kansas* OR<br>Kentucky* OR<br>Louisiana* OR<br>Maine* OR<br>Maryland* OR<br>Massachusetts* OR<br>Michigan* OR<br>Minnesota* OR<br>Mississippi* OR<br>Missouri* OR<br>Montana* OR<br>Nebraska* OR<br>Nevada* OR<br>"New Hampshire*" OR<br>"New Jersey*" OR<br>"New Mexico*" OR<br>"New York*" OR<br>"North Carolina*" OR<br>"North Dakota*" OR<br>Ohio* OR<br>Oklahoma* OR<br>Oregon* OR<br>Pennsylvania* OR<br>"Rhode Island*" OR<br>"South Carolina*" OR<br>"South Dakota*" OR	Arkansas* OR<br>California* OR<br>Colorado* OR<br>Connecticut* OR<br>Delaware* OR<br>Florida* OR<br>Georgia* OR<br>Hawai* OR<br>Idaho* OR<br>Illinois* OR<br>Indiana* OR<br>Iowa* OR<br>Kansas* OR<br>Kentucky* OR<br>Louisiana* OR<br>Maine* OR<br>Maryland* OR<br>Massachusetts* OR<br>Michigan* OR<br>Minnesota* OR<br>Mississippi* OR<br>Missouri* OR<br>Montana* OR<br>Nebraska* OR<br>Nevada* OR<br>"New Hampshire" OR<br>"New Jersey" OR<br>"New Mexico" OR<br>"New York" OR<br>"North Carolina" OR<br>"North Dakota" OR<br>Ohio* OR<br>Oklahoma* OR<br>Oregon* OR<br>Pennsylvania* OR<br>"Rhode Island" OR<br>"South Carolina" OR<br>"South Dakota" OR

For peer review only

	Nevada* [tiab] OR<br>New Hampshire* [tiab] OR<br>New Jersey* [tiab] OR<br>New Mexico* [tiab] OR<br>New York* [tiab] OR<br>North Carolina* [tiab] OR<br>North Dakota* [tiab] OR<br>Ohio* [tiab] OR<br>Oklahoma* [tiab] OR<br>Oregon* [tiab] OR<br>Pennsylvania* [tiab] OR<br>Rhode Island* [tiab] OR<br>South Carolina* [tiab] OR<br>South Dakota* [tiab] OR<br>Tennessee* [tiab] OR<br>Texas* [tiab] OR<br>Utah* [tiab] OR<br>Vermont* [tiab] OR<br>Virginia* [tiab] OR<br>Washington* [tiab] OR<br>West Virginia* [tiab] OR<br>Wisconsin* [tiab] OR<br>Wyoming* [tiab] OR<br>Washington D.C. [tiab] OR<br>District of Columbia [tiab])<br>)	Nevada* OR<br>"New Hampshire*" OR<br>"New Jersey*" OR<br>"New Mexico*" OR<br>"New York*" OR<br>"North Carolina*" OR<br>"North Dakota*" OR<br>Ohio* OR<br>Oklahoma* OR<br>Oregon* OR<br>Pennsylvania* OR<br>"Rhode Island*" OR<br>"South Carolina*" OR<br>"South Dakota*" OR<br>Tennessee* OR<br>Texas* OR<br>Utah* OR<br>Vermont* OR<br>Virginia* OR<br>Washington* OR<br>"West Virginia*" OR<br>Wisconsin* OR<br>Wyoming* OR<br>"Washington D.C." OR<br>"District of Columbia")<br>)	Indiana*.mp OR<br>Iowa*.mp OR<br>Kansas*.mp OR<br>Kentucky*.mp OR<br>Louisiana*.mp OR<br>Maine*.mp OR<br>Maryland*.mp OR<br>Massachusetts*.mp OR<br>Michigan*.mp OR<br>Minnesota*.mp OR<br>Mississippi*.mp OR<br>Missouri*.mp OR<br>Montana*.mp OR<br>Nebraska*.mp OR<br>Nevada*.mp OR<br>New Hampshire*.mp OR<br>New Jersey*.mp OR<br>New Mexico*.mp OR<br>New York*.mp OR<br>North Carolina*.mp OR<br>North Dakota*.mp OR<br>Ohio*.mp OR<br>Oklahoma*.mp OR<br>Oregon*.mp OR<br>Pennsylvania*.mp OR<br>Rhode Island*.mp OR<br>South Carolina*.mp OR<br>South Dakota*.mp OR<br>Tennessee*.mp OR<br>Texas*.mp OR<br>Utah*.mp OR<br>Vermont*.mp OR<br>Virginia*.mp OR<br>Washington*.mp OR<br>West Virginia*.mp OR<br>Wisconsin*.mp OR<br>Wyoming*.mp OR<br>District of Columbia.mp)	Tennessee* OR<br>Texas* OR<br>Utah* OR<br>Vermont* OR<br>Virginia* OR<br>Washington* OR<br>"West Virginia*" OR<br>Wisconsin* OR<br>Wyoming* OR<br>"Washington D.C." OR<br>"District of Columbia")<br>)	Tennessee* OR<br>Texas* OR<br>Utah* OR<br>Vermont* OR<br>Virginia* OR<br>Washington* OR<br>"West Virginia" OR<br>Wisconsin* OR<br>Wyoming* OR<br>"Washington D.C." OR<br>"District of Columbia")<br>)

For peer review only

			)		
**Adolescents:**	Adol* [tiab] OR<br>Young* [tiab] OR<br>Youth*[tiab] OR<br>Teen*[tiab] OR<br>Adolescent [MH] OR<br>Young Adult [MH]	Adol* OR<br>Young* OR<br>Youth* OR<br>Teen* OR<br>(MH "Adolescence+") OR<br>(MH "Pregnancy in Adolescence") OR<br>(MH "Young Adult")	Adol*.mp OR<br>Young*.mp OR<br>Youth*.mp OR<br>Teen*.mp OR<br>exp adolescent/ OR<br>young adult/	TITLE-ABS-KEY(<br>Adol* OR<br>Young* OR<br>Youth* OR<br>Teen*)	Adol* OR<br>Young* OR<br>Youth* OR<br>Teen*
**Qualitative research:**	Interview* [tiab] OR<br>Experience* [tiab] OR<br>Qualitative [tiab] OR<br>Theme* [tiab] OR<br>Thematic [tiab] OR<br>Focus group* [tiab] OR<br>Participatory [tiab] OR<br>Qualitative Research [MH:NOEXP]	Interview* OR<br>Experience* OR<br>Qualitative OR<br>Theme* OR<br>Thematic OR<br>“Focus group*” OR<br>Participatory OR<br>(MH "Qualitative Studies+")	Interview*.mp OR<br>Experience*.mp OR<br>Qualitative.mp OR<br>Theme*.mp OR<br>Thematic.mp OR<br>Focus group*.mp OR<br>Participatory.mp OR<br>qualitative research/ OR<br>qualitative analysis/	TITLE-ABS-KEY(<br>Interview* OR<br>Experience* OR<br>Qualitative OR<br>Theme* OR<br>Thematic OR<br>“Focus group*” OR<br>Participatory)	Interview* OR<br>Experience* OR<br>Qualitative OR<br>Theme* OR<br>Thematic OR<br>“Focus group” OR<br>“Focus groups” OR<br>Participatory

For peer review only

Preferred Reporting Items for Systematic reviews and Meta-Analyses extension for Scoping Reviews (PRISMA-ScR) Checklist

SECTION	ITEM	PRISMA-ScR CHECKLIST ITEM	REPORTED ON PAGE #
**TITLE**			
Title	1	Identify the report as a scoping review.	
**ABSTRACT**			
Structured summary	2	Provide a structured summary that includes (as applicable): background, objectives, eligibility criteria, sources of evidence, charting methods, results, and conclusions that relate to the review questions and objectives.	
**INTRODUCTION**			
Rationale	3	Describe the rationale for the review in the context of what is already known. Explain why the review questions/objectives lend themselves to a scoping review approach.	
Objectives	4	Provide an explicit statement of the questions and objectives being addressed with reference to their key elements (e.g., population or participants, concepts, and context) or other relevant key elements used to conceptualize the review questions and/or objectives.	
**METHODS**			
Protocol and registration	5	Indicate whether a review protocol exists; state if and where it can be accessed (e.g., a Web address); and if available, provide registration information, including the registration number.	
Eligibility criteria	6	Specify characteristics of the sources of evidence used as eligibility criteria (e.g., years considered, language, and publication status), and provide a rationale.	
Information sources*	7	Describe all information sources in the search (e.g., databases with dates of coverage and contact with authors to identify additional sources), as well as the date the most recent search was executed.	
Search	8	Present the full electronic search strategy for at least 1 database, including any limits used, such that it could be repeated.	
Selection of sources of evidence†	9	State the process for selecting sources of evidence (i.e., screening and eligibility) included in the scoping review.	
Data charting process‡	10	Describe the methods of charting data from the included sources of evidence (e.g., calibrated forms or forms that have been tested by the team before their use, and whether data charting was done independently or in duplicate) and any processes for obtaining and confirming data from investigators.	
Data items	11	List and define all variables for which data were sought and any assumptions and simplifications made.	
Critical appraisal of individual sources of evidence§	12	If done, provide a rationale for conducting a critical appraisal of included sources of evidence; describe the methods used and how this information was used in any data synthesis (if appropriate).	
Synthesis of results	13	Describe the methods of handling and summarizing the data that were charted.	

St. Michael's
Inspired Care. Inspiring Science.

For peer review only - http://bmjopen.bmj.com/site/about/guidelines.xhtml

SECTION	ITEM	PRISMA-ScR CHECKLIST ITEM	REPORTED ON PAGE #
**RESULTS**			
Selection of sources of evidence	14	Give numbers of sources of evidence screened, assessed for eligibility, and included in the review, with reasons for exclusions at each stage, ideally using a flow diagram.	
Characteristics of sources of evidence	15	For each source of evidence, present characteristics for which data were charted and provide the citations.	
Critical appraisal within sources of evidence	16	If done, present data on critical appraisal of included sources of evidence (see item 12).	
Results of individual sources of evidence	17	For each included source of evidence, present the relevant data that were charted that relate to the review questions and objectives.	
Synthesis of results	18	Summarize and/or present the charting results as they relate to the review questions and objectives.	
**DISCUSSION**			
Summary of evidence	19	Summarize the main results (including an overview of concepts, themes, and types of evidence available), link to the review questions and objectives, and consider the relevance to key groups.	
Limitations	20	Discuss the limitations of the scoping review process.	
Conclusions	21	Provide a general interpretation of the results with respect to the review questions and objectives, as well as potential implications and/or next steps.	
**FUNDING**			
Funding	22	Describe sources of funding for the included sources of evidence, as well as sources of funding for the scoping review. Describe the role of the funders of the scoping review.	

JBI = Joanna Briggs Institute; PRISMA-ScR = Preferred Reporting Items for Systematic reviews and Meta-Analyses extension for Scoping Reviews.

* Where *sources of evidence* (see second footnote) are compiled from, such as bibliographic databases, social media platforms, and Web sites.

† A more inclusive/heterogeneous term used to account for the different types of evidence or data sources (e.g., quantitative and/or qualitative research, expert opinion, and policy documents) that may be eligible in a scoping review as opposed to only studies. This is not to be confused with *information sources* (see first footnote).

‡ The frameworks by Arksey and O'Malley (6) and Levac and colleagues (7) and the JBI guidance (4, 5) refer to the process of data extraction in a scoping review as data charting.

§ The process of systematically examining research evidence to assess its validity, results, and relevance before using it to inform a decision. This term is used for items 12 and 19 instead of "risk of bias" (which is more applicable to systematic reviews of interventions) to include and acknowledge the various sources of evidence that may be used in a scoping review (e.g., quantitative and/or qualitative research, expert opinion, and policy document).

From: Tricco AC, Lillie E, Zarin W, O'Brien KK, Colquhoun H, Levac D, et al. PRISMA Extension for Scoping Reviews (PRISMAScR): Checklist and Explanation. Ann Intern Med. 2018;169:467–473. doi: 10.7326/M18-0850.

For peer review only
